# Kv3-Expressing Cells Present More Elaborate N-Glycans with Changes in Cytoskeletal Proteins, Neurite Structure and Cell Migration

**Published:** 2023-07-27

**Authors:** M Kristen Hall, Asif Shajahan, Cody J Hatchett, Adam P Burch, Parastoo Azadi, Ruth A Schwalbe

**Affiliations:** 1 Department of Biochemistry and Molecular Biology, East Carolina University, USA; 2 Complex Carbohydrate Research Center, University of Georgia, USA

**Keywords:** Kv3 channels, N-glycosylation, Cytoskeletal proteins, Cell morphology, Glycans, Protein folding, Cell motility

## Abstract

The cues contained by N-glycans relay the quality, cellular destination, and interactions of proteins, thereby, impacting cellular physiology. Voltage-gated K^+^ (Kv3) channels have two conserved N-glycosylation sites which are vital for Kv3 channel activity, and primary motor neuron development. Our previous studies showed that the parental (NB_1) and N-glycan mutant (NB_1(-Mgat1), NB_1(-Mgat2), and NB_1(-Mgat3)) Neuroblastoma (NB) cell lines have compromised N-acetylglucosaminyltransferase activities: GnT-I, GnT-II, or GnT-III. Herein, we stably expressed Kv3.1b in the parental and engineered N-glycan mutant Neuroblastoma (NB) cell lines to examine how changes in N-glycans alter the cytoskeleton, and subsequently cellular properties. MALDI-TOF MS verified that the parental and mutant cell lines had different N-glycan profiles. When Kv3.1b was expressed in NB cells with an intact Mgat1, the N-glycan population had more complex N-glycans with increased branching. Further NB cells with an intact Mgat2 had higher and lower levels of hybrid and oligomannose N-glycans, respectively. N-Glycan populations changed cytoskeletal protein abundancies and cell morphology. Moreover, all Kv3.1b-expressing cells, except NB_1(-Mgat2), had changed levels of F-actin, neurofilament and vimentin, along with modified neurite formation. In all cases, migratory rates were enhanced when cells expressed Wt Kv3.1b. Glycan populations and glycans attached to Kv3.1b altered spatial arrangement in membranes and both ER folding and transport proteins were not increased by expression of unglycosylated Kv3.1b. Kv3.1b expression in NB cells alters N-glycan populations and mediates adjustments in cytoskeletal proteins, and thereby cell morphology and cell migration, supporting roles in neuronal development and maintenance.

## Introduction

Defects in the N-glycosylation pathway result in conditions known as Congenital Disorders of Glycosylation (CDG) [1]. Although CDGs affect different organ systems, the neurological impact is detrimental and is noted in most types [2]. Glycosylation is a post translational modification necessary for protein development, function, and stability. As such, glycosylation is paramount in the development of an organism. The glycosylation process occurs via the addition of monosaccharides to proteins [2]. N-glycosylation is characterized by the addition of an oligosaccharide to a protein at an asparagine residue via an N-glycosidic bond. The three major types of N-glycans are classified as oligomannose, hybrid, and complex [3]. N-glycans are composed of a conserved pentasacchride region, and processing occurs via the actions of N-Acetylglucosaminyltransferases (GnTs), which are enzymes encoded by MGAT genes. GnTs add a GlcNAc residue to the conserved pentasaccharide yielding the various types of N-glycans. GnT-I activity promotes the transition from oligomannose to hybrid type N-glycans, while GnT-II converts hybrid glycans to complex type N-glycans. GnT-III adds a bisecting GlcNAc residue and results in termination of glycan processing [3]. For this reason, evaluation of how modifications in N-glycan content of cells, including processing of N-glycosylated proteins (e.g., voltage-gated ion channels), is a critical area of research for therapeutic development of CDG and neurodegenerative diseases.

Voltage-gated Kv3 channels are a family of potassium ion channels that are associated with high-frequency firing neurons because of their unique depolarized activation range and rapid activation/deactivation rates [4–6]. In addition to the traditional conducting role of ion channels, Kv3 also regulates cell signaling, cell adhesion, and cytoskeleton networks through its non-conducting roles [7]. The conducting and non-conducting functions of Kv3 are essential for proper neurodevelopment as alterations in the activity or expression of Kv3 has been linked to various neurodevelopmental disorders [8,9] and cancer [10]. N-glycosylation has been shown to influence both conducting and non-conducting roles of Kv3 channels. Removal of one or both conserved N-glycosylation sites of the α-subunit of Kv3.1, along with replacement of complex N-glycans of Kv3.1 with oligomannose or hybrid, was shown to reduce cell migration, encourage localization of Kv3 in the cell body rather than the neurites, hinder the opening and closing kinetics of Kv3 channels, and reduce neurite length in rat neuroblastoma cells [11–13]. Furthermore, Kv3 channels with only one of the two conserved N-glycosylation sites occupied resulted in shortened axons and reduced synapse formation of motor neurons in zebrafish [13].

Protein folding and quality control within the Endoplasmic Reticulum (ER) are regulated by molecular chaperones. These ER chaperones are crucial to the regulation/maintenance of ER stress response, intracellular signaling, proper protein folding and Ca^2+^ storage [14]. Proper N-glycosylation of proteins is regulated by two different families of chaperones: Heat Shock Proteins (HSP), which interact with unglycosylated and glycosylated proteins; and lectins that interact with incompletely folded glycosylated proteins [15]. Grp94 and Bip belong to the HSP family of ER chaperones and they are responsible for directing misfolded N-glycosylated proteins for degradation or correction [16,17]. Likewise, Calreticulin belongs to the lectin family and is a calcium binding protein that assists in proper protein folding [18]. Calreticulin acts within the calnexin/calreticulin cycle which dictates the shuttling of glycosylated proteins from the ER to the Golgi apparatus [19]. ER function also involves ER transport proteins. The kinesin superfamily proteins (KIFs) are involved in bidirectional mobility from the ER, and neuronal cells have an advanced transport system [20]. For instance, KIF5 (kinesin-1) is the most abundant microtubule motor protein and is vital for axonal transport [20].

Cytoskeletal proteins, such as actin filaments, intermediate filaments and microtubules, help to form the shape of the cell, assist in movement, and play fundamental roles in neuronal development [21]. The function and role of actin is highly dependent upon its ability to self-polymerize into actin filaments (F-actin) or depolymerize into Globular Actin (G-actin) [22]. Vimentin, an intermediate filament, has been linked to neurons undergoing differentiation and neurite extension, and is thought to be associated with neuronal damage in Alzheimer’s patients [23]. Neurofilament, like vimentin, is involved in neuronal development, but is associated with more differentiated/mature neurons [24–26]. In fact, Yabe et al. [25] describes a transition from vimentin to neurofilament as part of neuronal development. Here we examine Neurofilament-Light chain(NF-L), which has been implicated as a biomarker in the progression of Parkinson’s disease [27,28] and other neurodegenerative diseases [29].

Our goal of this paper was to determine whether Kv3.1b expression in NB cell lines with different N-glycan populations, could influence the N-glycosylation pathway, and consequently, cell structure and function in cultured Neuroblastoma (NB) cells, a neuronal-derived cell model. Previously, ESI-MS studies showed that our engineered N-glycan mutant cell lines NB_1(-Mgat1), NB_1(-Mgat2) and NB_1(-Mgat3) have compromised N-acetylglucosaminyltransferase (GnT-I), (GnT-II), or (GnT-III) activities, relative to the parental cell line [30]. Here MALDI-TOF MS was employed to compare N-glycan populations in non transfected NB cell lines, and NB cell lines stably transfected with wild type (Wt) Kv3.1b. Western blotting was utilized to examine N-glycosylation of Kv3.1b in NB cell lines transfected with Kv3.1b. This technique was also employed to compare expression levels of ER chaperone (Grp94 and calreticulin) and ER transport (KIF5B) proteins, along with proteins of the cytoskeletal network (F-actin, vimentin, and neurofilament-L), in parental and glycosylation mutant NB cell lines without and with expression of Wt Kv3.1b. Changes in cytoskeleton protein levels supported modifications in neurite structure and cell mobility. Taken together, we postulate that changes in the N-glycosylation pathway and Kv3.1b levels mediate adaptions to neurite structure and cell migration through cytoskeletal and ER proteins which are needed for neuronal development and maintenance.

## Materials and Methods

### Cell lines, cell culture and cell transfection

NB_1 is a clonal cell line derived from Rat B35 Neuroblastoma (NB) (American Type Culture collection, Manassas, VA, USA) and was the parental cell line utilized to engineer the glycosylation mutant cell lines (NB_1(-Mgat1) and NB_1(-Mgat2), and NB_1(-Mgat3) via CRISPR/Cas9 technology [30]. The different N-glycan populations in the various cell lines were identified via ESI-MS supporting at least partial knock-down of the mutant cell lines [30]. The construction of the NB_1, NB_1(-Mgat1) and NB_1(-Mgat2) cell lines stably expressing Wt (wild type) or N220/229Q Kv3.1b were previously described [12], and NB_1(-Mgat3)_Wt Kv3.1b and NB_1(-Mgat3)_N220/229Q Kv3.1b cell lines were completed in a similar manner. In all cases, an EGFP tag is fused to the C-terminus of Kv3.1b. All cells were maintained in DMEM containing 10% FBS, 50U/mL penicillin, and 50μg/mL streptomycin at 37° in a 5% CO_2_ atmosphere.

### Total membrane protein fractions and whole cell lysates of NB cells

Harvesting of total membranes was performed via ultracentrifugation as described previously [12]. Whole cell lysates were collected in RIPA buffer (PBS, 1% Triton X-100, 0.5% sodium deoxycholate, 0.1% SDS) containing protease inhibitor cocktail set III (EMD Biosciences San Diego, CA, USA) [11]. Samples were reduced/denatured for western blotting by addition of SDS-PAGE sample buffer. Samples were stored at −20° until needed.

### F-actin and G-actin isolation

Cells were resuspended in LAS2 buffer (50mM Pipes, 50mM NaCL, 5mM MgCl_2_, 5mM EGTA, 5% Glycerol, 0.1% Nonidet P40, 0.1% TritonX100, 0.1% Tween 20, 0.1% 2-mecraptoethanol, 0.001% Antifoam C, 1mM ATP, 1mM Tris pH 7.5, and protease inhibitor cocktail set III (EMD Biosciences San Diego, CA, USA) by scraping and gentle pipetting, and then incubated at 37° for 10 min. An aliquot of resuspended cell solution was transferred to a clean Eppendorf tube and spun at 2,000rpm for 5 minutes. The remaining cell solution was stored at −20° for future use. The supernatant was transferred to an ultracentrifuge tube and then spun at 100,000× g for 1h at 37° using a Beckman Coulter Optima^™^ MAX-XP ultracentrifuge (Beckman Coulter, Brea, CA). The supernatant, containing G-actin, was transferred to an Eppendorf tube while the pellet, containing the F-actin, was resuspended in 100μL depolymerization buffer (5mM TrisHCl, 8M urea). Samples were stored in reducing buffer containing 2% 2-mercaptoethanol at −20° for future use.

### Western blotting

Total membranes from Kv3.1b transfected cells were separated on 10% SDS gels at 20mA and transferred to PVDF membranes (Millipore, Billercia, MA, USA) at 0.25 A, as previously described [31]. Subsequently, transferred membranes were incubated with anti-Kv3.1b (NeuroMab, Davis, CA, USA) antibody and visualized with NBT/BCIP. Multiplex fluorescent western blotting was used for quantification of the protein of interest. In short, whole cell lysate samples from Kv3.1b transfected and non-transfected cells were electrophoresed on Any kD^™^ or 10% SDS gels (Biorad, Hercules, CA). Separated proteins were transferred to PVDF membranes and were incubated in EveryBlot Blocking Buffer, primary, and secondary antibodies, and then imaged using a ChemiDoc MP imaging system which allowed visualization of two proteins simultaneously using fluorescent antibodies. Primary antibodies include: BiP, vimentin, calreticulin, Grp94, neurofilament-L (NF-L) and KIF5B from (Cell Signaling Technology, Danvers, MA) and rhodamine conjugated β-actin (Biorad, Hercules, CA). Secondary antibodies (Biorad, Hercules, CA) include anti-mouse IgG starBright Blue 700, anti-rabbit IgG starBright Blue 700, anti-mouse IgG starBright Blue 520, anti-rabbit IgG starBright Blue 520. Image Lab software (Biorad, Hercules, CA) was used for data acquisition and quantification of the immuno-band of interest. The protein of interest and β-actin were simultaneously analyzed in a single sample on the same blot which allowed for correction of sample load and the transfer of proteins from gel to membrane. The ratio of immuno-band intensities for the protein of interest and β-actin were determined in a single sample. The means of ratio values for a given protein from at least 3 separate loads were determined, and thus plotted relative to β-actin.

### N-Glycomics method

Glycan profiling was performed like previously described (Shajahan et al., 2020) Total membrane protein fractions extracted from NB cells were diluted with 100μl of 50mM ammonium bicarbonate buffer. Subsequently, 25μL of 25mM Dithiothreitol (DTT) was added and incubated at 50 °C for 30min. The samples were desalted by centrifugation via Amicon centrifuge filters (MilliporeSigma, Cat. No. UFC501096). Desalted samples were then ultrasonicated to dissolve the proteins. 5μL of PNGaseF (New England Biolabs, Cat. No. P0709L) was added to desalted ultrasonicated samples followed by incubation at 37 °C for 48h to release the N-glycans. Addition of water quenched the reaction and permethylated N-glycans were extracted with dichloromethane. The dichloromethane layer was rinsed four times with water and dried via evaporation by nitrogen gas. Permethylated N-glycans were redissolved in 20μL of methanol. 1μL of a mixture of 1μL of the permethylated N-glycans and 1μL of DHB (dihydrobenzoic acid) matrix (10mg/mL in 1:1 methanol-water was spotted on a MALDI plate and analyzed by MALDI-TOF-MS (ABSciex MALDI-TOF/TOF 5800 mass spectrometer). GlycoworkBench software was used to identify the N-glycan structures based on precursor masses (sodium adduct) obtained by MALDI-TOF-MS and common mammalian biosynthetic pathway [30].

### Cell morphology assay

Differential Interference Contrast (DIC) images were acquired using an Olympus IX-71 microscope (Olympus, Center Valley, PA, USA) with an Apo 60×1.45 objective. Cells were plated at low density on poly-lysine coated culture dishes (Mat-Tek, Ashland, MA, USA) and incubated for 18 hours prior to imaging. Using Image J Software, the number of neurites and neurite length were determined. Further, the number of neurites with filopodia or lamellipodia, plus neurites with both filopodia and lamellipodia were counted, and percent of structure(s) was determined for each cell line.

### Wound healing assay

Cell migration assays were performed as previously described [11]. In brief, non-transfected and Kv3.1b transfected NB cells were plated on 60 mm cell bind dishes (Corning, Corning, NY, USA) and allowed to grow to confluency. Culture medium was then aspirated, and cell monolayer was disrupted using a beveled 200μl pipette tip (Fisher Scientific, Suwanee, GA, USA). Cellular debris was removed from the plate by washing dish two times with DMEM. DMEM was added to plate and images were obtained at 0 and 19 hour time points (4x objective) using an Olympus IX73 Microscope (Olympus, Shinjuku City, Tokyo, Japan). Wound size was measured using AdobePhotoshop (Adobe Inc., San Jose, California, USA). Initial wound size (0 hour) and final wound size (19 hour) was determined, and percent wound closure was calculated using the following equation: $\frac{\mathrm{initial}\;\mathrm{wound}\;\mathrm{size}\;-\;\mathrm{final}\;\mathrm{wound}\;\mathrm{size}}{\mathrm{initial}}\times 100$.

The mobility due the N-glycan was calculated using the following equation:

$$\frac{\mathrm{glycosylated}\;\mathrm{Kv}3.1b\;\mathrm{cell}\;\mathrm{mobility}\;-\;unglycosylated\;Kv3.1b\;\mathrm{mobility}}{unglycosylated\;Kv3.1b\;\mathrm{mobility}}$$

### TIRF microscopy

Microscopy images were captured and analyzed as previously described [12]. In brief, Kv3.1b-EGFP transfected cells were seeded at low density on 35mm poly-L-lysine glass bottom dishes followed by 18hrs incubation. Images were acquired using an Olympus IX-71 microscope (Olympus, Center Valley, PA, USA) with an Apo 60× 1.45 objective with all laser controls mediated by Cell^TIRF 1.1 with Metamorph software. Cell excitation was mediated via a 488nm Argon laser with 1000ms exposure time. The percent of protein localized to the neurites was calculated using ImageJ software. The percent of Kv3.1b in the neurites was determined by dividing area of fluorescent intensity of the neurites by area of fluorescent intensity of the entire cell.

### Confocal microscopy

Kv3.1b-EGFP transfected cells were plated on 35mm poly-L-lysine glass bottom dishes. Imaging was carried out after 18 hours post incubation using a Carl Zeiss LSM 700 Microscope using either Plan-Apochromat 40x/1.40 oil immersion DIC M27 objective. Optical sectioning was performed at 1μm increments to generate image stacks. Excitation of the tagged Kv3.1b protein was performed using a 488nm solid state laser with a frame size of 2048×2048 pixels, 8-bit image depth and a scanning speed of 6. Image stacks were compressed and the percent of Kv3.1b in the neurites was calculated using ImageJ software. Measurements were obtained for both the entire cell and the neurites individually. The percent of Kv3.1b in the neurites was determined by dividing area of fluorescent intensity of the neurites by area of fluorescent intensity of the entire cell. In all cases, bright field images were obtained to verify neurite and some areas.

### Data analysis

Adobe Photoshop was utilized for Western blot pictures. Western blots were quantified using Bio-Rad Image Lab 6.1. Graphics and statistics were obtained using Origin 9.55. Data are presented as the mean S.E. where n signifies the number of observations. Statistical comparison between two groups was completed using Student’s t-test. One-way ANOVA with Bonferroni adjustments was implemented when comparing more than two groups, unless otherwise denoted.

## Results

### Expression of glycosylated and unglycosylated Kv3.1b NB cells

**Figure 1:**
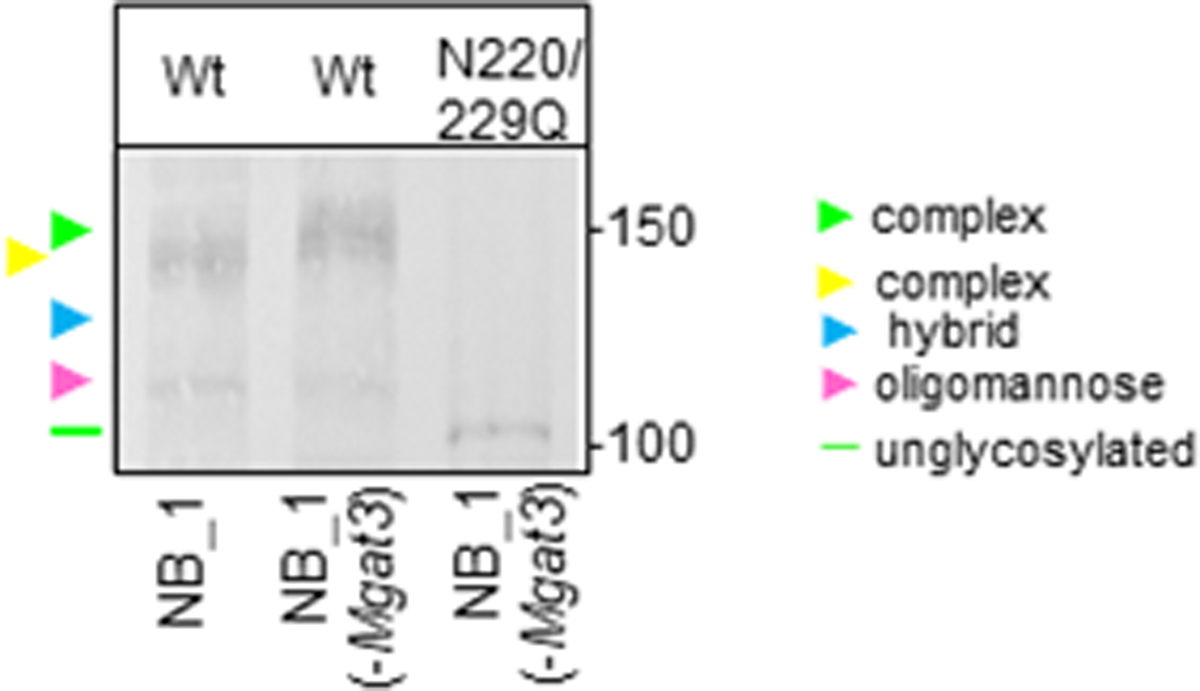
N-Glycosylation processing of Wt. Kv3.1b is changed in NB_1(-Mgat3) cells relative to the parental NB_1 cell line. Western blot of total membranes of NB_1 and NB_1(-Mgat3) stably expressing wild type (Wt) Kv3.1b or N220/229Q Kv3.1b. Molecular weight standards in kDa: 150, 100. Arrowheads denote glycosylated Kv3.1b protein with complex (green and yellow arrowheads); oligomannose (pink), hybrid (blue) types of N-glycans. Green line denotes glycosylated Kv3.1 protein.

Herein we investigated whether expression of Wt Kv3.1b in rat parental (NB_1) and N-glycosylation mutant NB_1 cell lines could alter N-glycan populations and cellular properties. The mutant cell lines (NB_1(-Mgat1), NB_1(-Mgat2) and NB_1(-Mgat3)) have different N-glycan profiles due to lowered activity of a N-acetylglucosaminyltransferase (GnT-I, -II, or -III) [30]. GnT-I catalyzes the conversions of oligomannose to hybrid while GnT-II converts hybrid to complex [3]. GnT-III (encoded by Mgat3) catalyzes the addition of bisecting GlcNAc residues to hybrid and complex N-glycans to create N-glycans with bisecting GlcNAc residues [3,32]. Moreover, the presence of a bisecting GlcNac residue terminates processing of N-glycans. Western blotting of total membrane proteins from the N-glycosylation mutant and parental cell lines expressing Wt Kv3.1b protein showed that the glycosylated Kv3.1b protein (green arrowhead) expressed in NB_1(-Mgat3) had a slower electrophoretic mobility compared to Kv3.1b (yellow arrowhead) in NB_1 (Figure 1). This is expected, if the activity of GnT-III was reduced, since the addition of a bisecting GlcNAc prevents processing of the N-glycan. In both cases, the faint lower band denotes oligomannose-containing Kv3.1b glycoprotein (pink arrowhead). When the unglycosylated (N220/229Q) Kv3.1b protein was expressed in NB_1(-Mgat3) cells, N220/229Q Kv3.1 (green line) migrated much faster, as expected. Based on past studies, the N-glycan types attached to glycosylated Kv3.1b in NB_1, NB_1(-Mgat2), and NB_1(-Mgat1) are complex [11], hybrid (blue arrowhead) [12], and oligomannose [11], respectively. Taken together, the results suggest that N-glycans attached to Kv3.1b in NB_1(-Mgat3) cells are of complex type but different from those attached to Kv3.1b in NB_1 cells. For instance, the increased mobility of glycosylated Kv3.1b expressed in NB_1(-Mgat3) cells may have complex N-glycans which are more branched and/or greater extension of the antenna.

### Expression of Wt Kv3.1b in neuronal-derived cells enhanced processing of N-glycans

**Figure 2:**
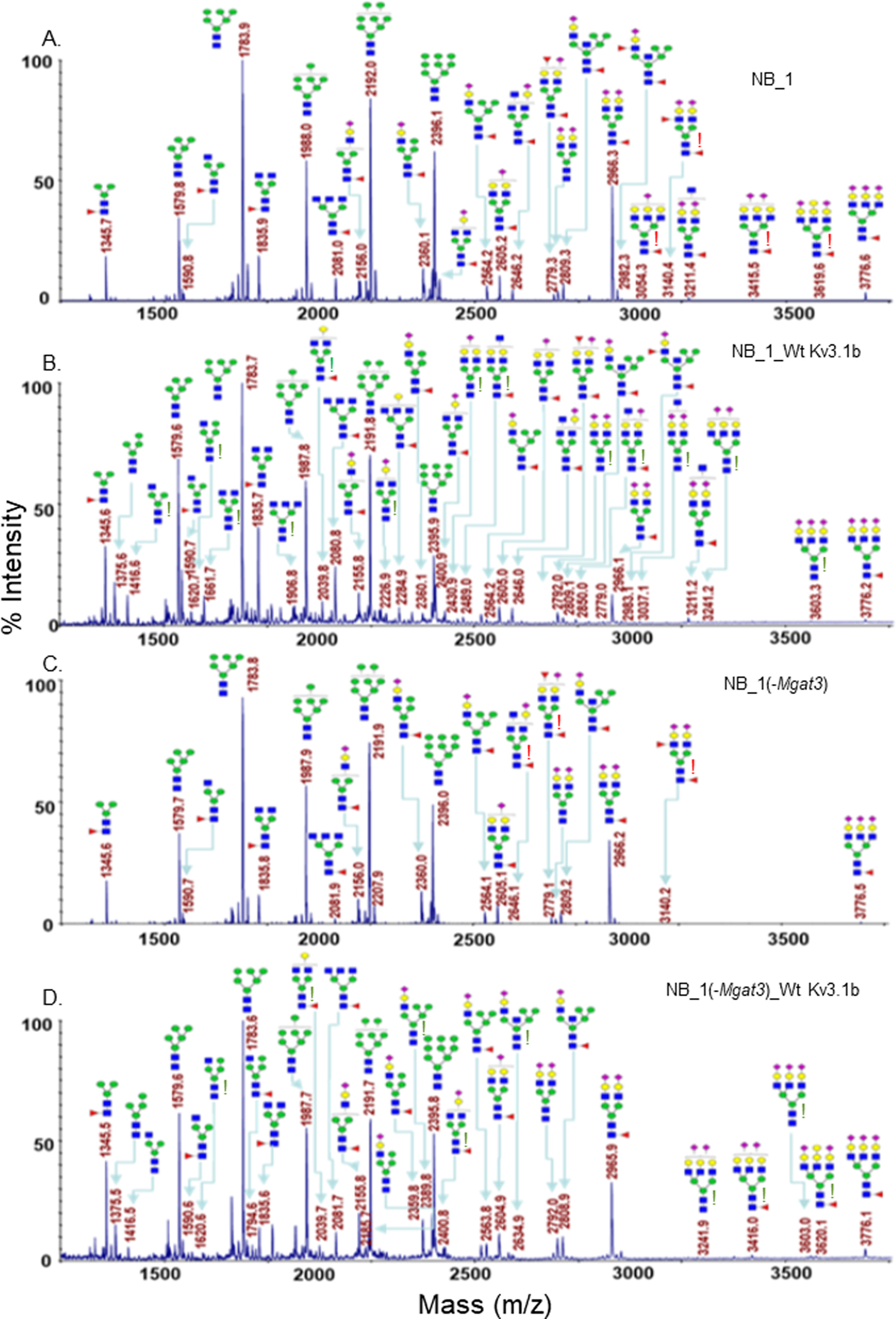
Comparison of MALDI-TOF MS profiles of the permethylated N-glycans derived from NB cell lines. NB cell lines without, NB_1 (A) and NB_1(-Mgat3) (C), and with, NB_1_Wt Kv3.1b (B), and NB_1(-Mgat3) Wt Kv3.1b (D), glycosylated Kv3.1b expression. Data were obtained from dichloromethane extracted permethylated N-glycans with molecular ions present in sodiated form ([M + Na]+). Red exclamation points (A, C) signify N-glycans found only in NB cell lines not expressing Kv3.1b. Green exclamation points (B, D) denote increased N-glycans detected in NB cell lines expressing Kv3.1b.

**Figure 3:**
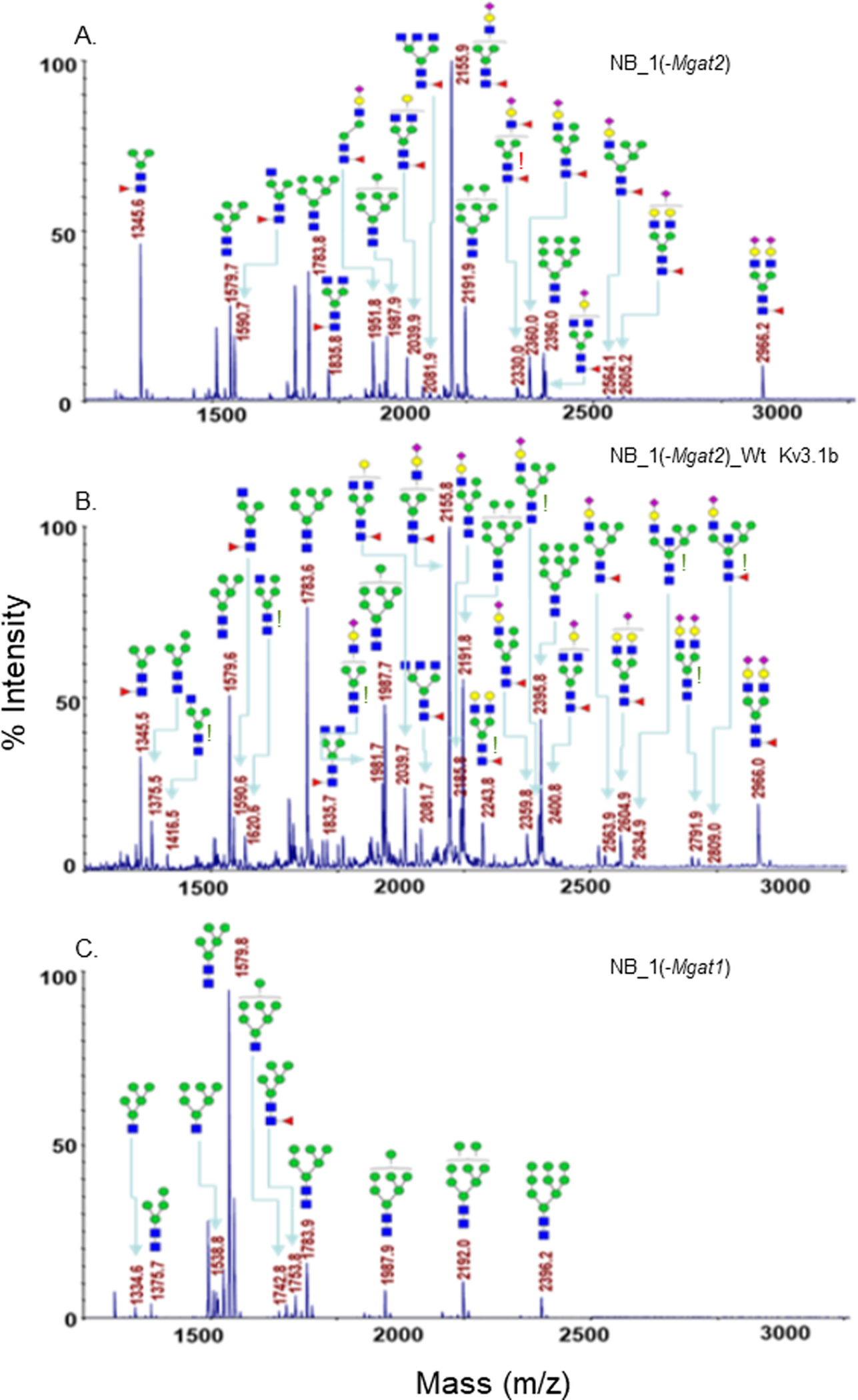
Glycan profiles of NB cell lines without an intact Mgat1 or Mgat2. MALDI- TOF MS profiles of permethylated N-glycans derived from NB_1(-Mgat2) (A), NB_1(-Mgat2)_Wt Kv3.1b (B) and NB_1(-Mgat1) (C) cell lines. Data were obtained from dichloromethane extracted permethylated N-glycans and all molecular ions present in sodiated form ([M + Na]+). Red exclamation points (A) represent N-glycans detected only in the NB cell lines not expressing Kv3.1b. Green exclamation points (B) signify increases in the NB cell lines expressing Kv3.1b.

To identify specific glycan structures, isolated permethylated N-glycans of the parental and N-glycosylation mutant cell lines without and with Wt Kv3.1b were analyzed by MALDI-TOF MS. Mass spectra of N-glycan structures from NB_1 (Figure 2A), NB_1_Wt Kv3.1b (Figure 2B), NB_1(-Mgat3) (Figure 2C), NB_1(-Mgat3)_Wt Kv3.1b (Figure 2D), NB_1(-Mgat2) (Figure 3A), NB_1(-Mgat2)_Wt Kv3.1b (Figure 3B) and NB_1(-Mgat1) (Figure 3C) cell lines. NB_1, NB_1(-Mgat2) and NB_1(-Mgat3) cell lines contained detectable levels of oligomannose, hybrid, and complex types of N-glycans while only oligomannose N-glycans were detected in the NB_1(-Mgat1) cell line. Since the NB_1(-Mgat1) cell line had only oligomannose type N-glycans, N-glycan profiling was not conducted for NB_1(-Mgat1) cells stably transfected with Wt Kv3.1b. Two more spectra for NB_1 cells and the relative abundancies of each of the glycan structures from all spectra are shown in the supplementary section. Of interest, the N-glycan profiles appeared to have raised levels of complex and hybrid types of N-glycans in NB_1, NB_1(-Mgat3), and NB_1(-Mgat2) expressing Kv3.1b. The red exclamation mark signifies N-glycans that were solely observed in the nontransfected cell lines while the green exclamation marks denote N-glycans that were increased when Kv3.1b was expressed in the cell lines. Thus, these changes in the levels of N-glycan structures of the various cell lines support that expression of Kv3.1b in NB_1, NB_1(-Mgat2), and NB_1(-Mgat3) increase the levels of complex and hybrid N-glycans.

**Figure 4:**
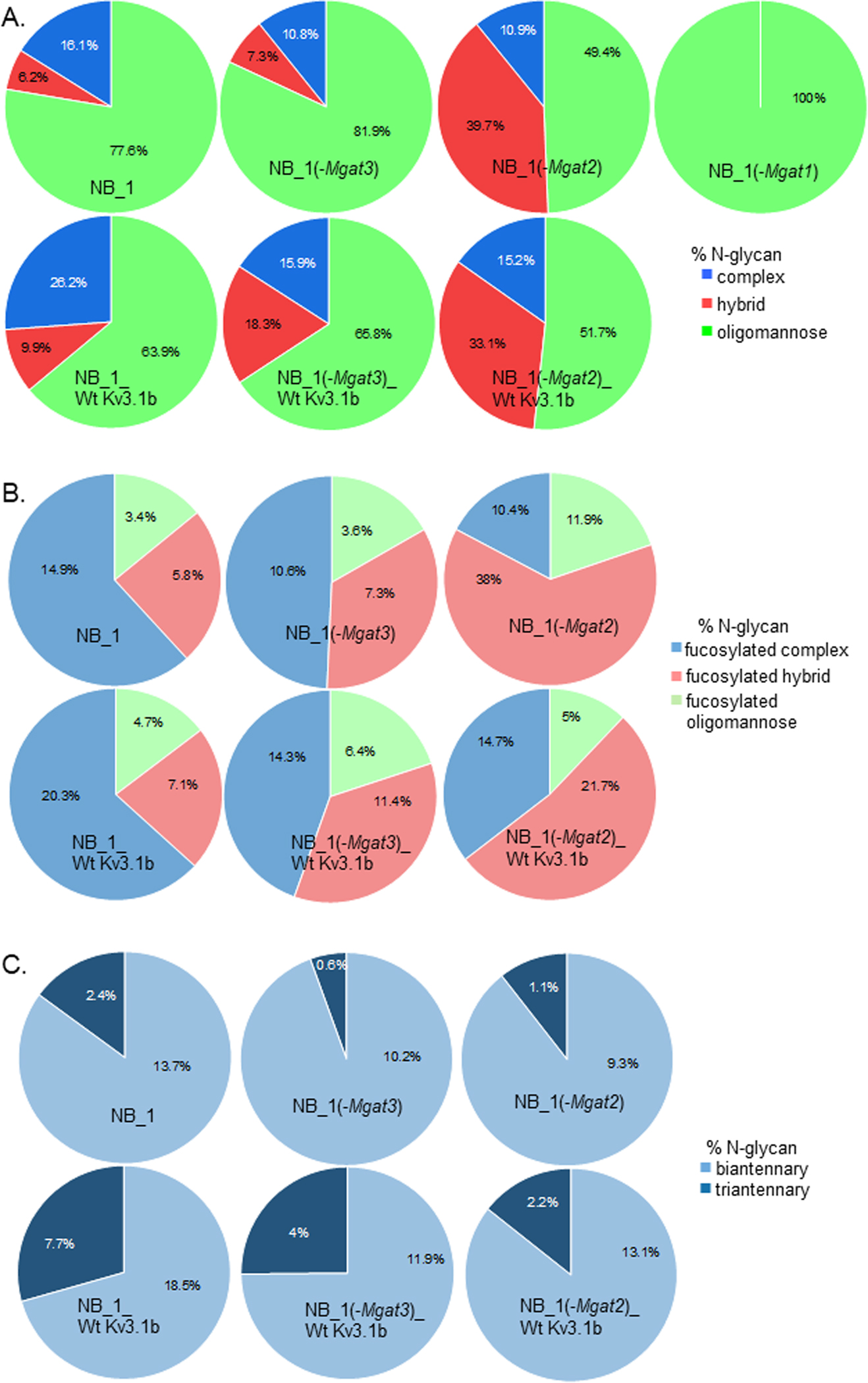
Quantification of MALDI-TOF MS profiles of permethylated N-glycans. Quantification of N-glycan types expressed in NB_1, NB_1 (-Mgat3), NB_1 (-Mgat2), and NB_1 (-Mgat1) cell lines (A, top row) and NB_1, NB_1 (-Mgat3), and NB_1 (- Mgat2) stably expressing Kv3.1b (A, bottom row). Fucosylated N-glycans by type in NB_1, NB_1 (-Mgat1), NB_1 (-Mgat2), and NB_1 (-Mgat3) cell lines (B, top row) and NB_1, NB_1 (-Mgat3), NB_1 (-Mgat2) stably expressing Kv3.1b (B, bottom row). Percent biantennary (light blue) and tri antennary (dark blue) N-glycans in NB_1, NB_1 (-Mgat3), NB_1 (-Mgat2), NB_1 (-Mgat1) cell lines (C, top row) and NB_1, and NB_1 (- Mgat3), NB_1 (-Mgat2) stably expressing Kv3.1b (C, bottom row).

The pie graphs show the levels of complex (blue), hybrid (red) and oligomannose (green) types of N-glycans in each of the NB cell lines (Figure 4A). When NB cells were stably transfected with glycosylated Kv3.1b, both NB_1_Wt Kv3.1b and NB_1(-Mgat3)_Wt Kv3.1b cell lines increased levels of complex and hybrid types while the oligomannose type decreased. The NB_1(-Mgat2)_Wt Kv3.1b cell line also increased the level of complex type N-glycan which was accompanied with a decrease in hybrid type N-glycans. Fucosylation of all types of N-glycans were increased in Kv3.1b-expressing NB_1 and NB_1(-Mgat3) cells while only fucosylated complex N-glycans were increased in NB_1(-Mgat2) cells expressing Wt Kv3.1b (Figure 4B). The branching of the complex type of N-glycans were increased in Kv3.1b-expressing cells (Figure 4C). Although the levels of both bi- and tri-antennary complex N-glycans were higher in all three cell lines expressing Wt Kv3.1b, there was a much larger increase in the levels of tri-antennary complex N-glycans. Moreover, the increase was highest in the NB_1(-Mgat3)_Wt Kv3.1b cell line. A consistent trend in sialylation of N-glycans without and with Kv3.1b was unobserved as sialylation decreased for complex and hybrid N-glycans from NB_1 and NB_1(-Mgat2), respectively, while sialylated N-glycans were unchanged for NB_1(-Mgat2). Thus, these results indicate that Kv3.1b-expressing cells raise the level of complex type N-glycans, along with increased branching, in three independent NB cell lines.

### Types of N-glycan and Kv3.1b expression influence neurite structure

**Figure 5:**
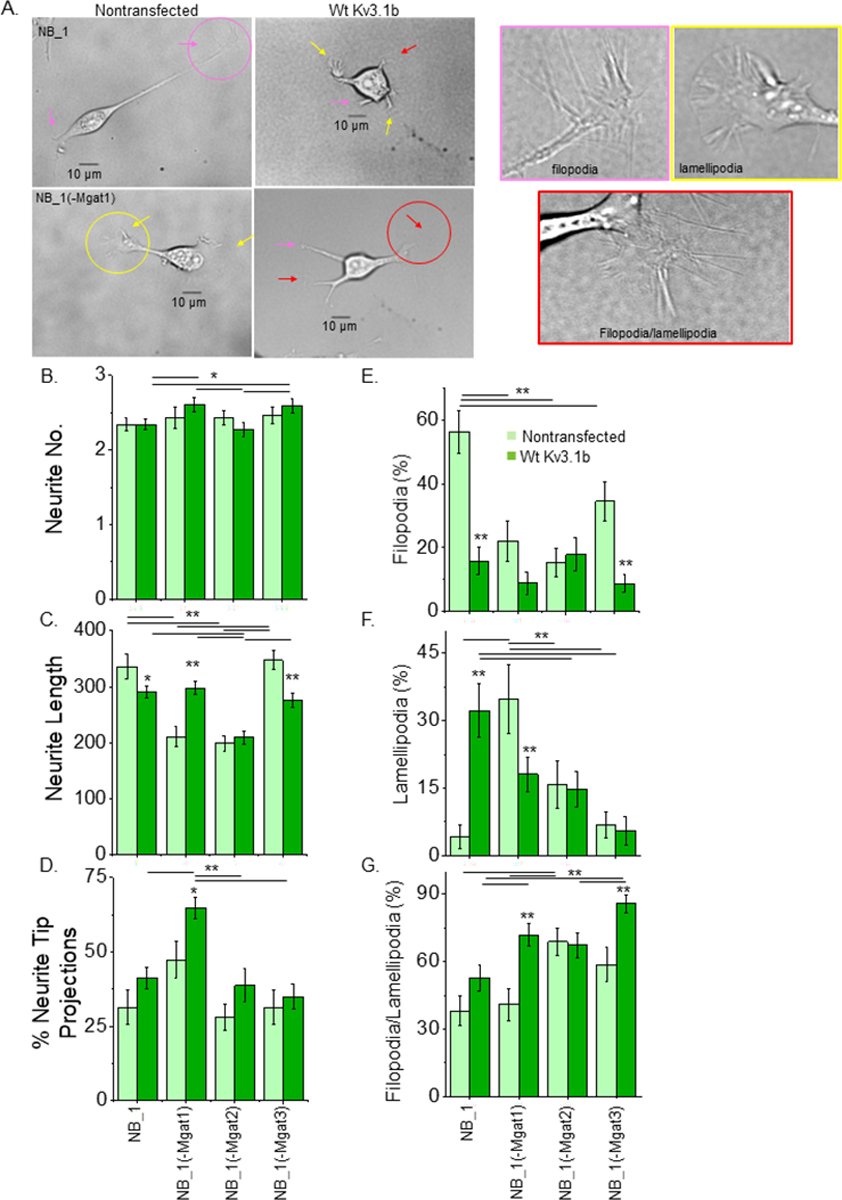
Impact of N-glycans and Kv3.1b on cell morphology. Images of NB_1 (upper row) and NB_1(-Mgat1) (lower row) non-transfected (left panel) or expressing glycosylated Kv3.1b (right panel) (A). Yellow, pink, and red arrows and circles correspond to legend indicating structure type. Images encased in yellow, pink and red boxes are magnified (3x) and correspond to encircled areas to highlight the various neurite structures analyzed. Quantification of neurite number (B; n≥20) length (C; n≥52), and % neurite tip protrusions (D; n≥52). Further analysis categorizing types of neurite tip protrusions as % filopodia (E; n≥23), % lamellipodia (F; n≥23), and % with both filopodia and lamellipodia (G; n≥23). Data are presented as mean ± SEM and were all compared by one-way ANOVA with Holm-Bonferroni mean comparison (*p < 0.1, **p < 0.05).

The morphology of the NB_1 cells consisted of a soma, one to several projections (neurites) from the cell body and projections from the tips of neurites (neurite tip projections) (Figure 5A). Arrows point to the various neurite tip projections. Neurite tips enclosed with circles were increased by 3-fold magnification (corresponding color boxes, right panels) to clearly show filopodia (pink), lamellipodia (yellow) and a mix of filopodia/lamellipodia (red). The number of neurites per cell were relatively similar in the parental and N-glycosylation mutant NB_1 cell lines (Figure 5B). However, differences could be detected when the cells were stably transfected with glycosylated Kv3.1b. Both NB_1(-Mgat1)_Wt Kv3.1b and NB_1(-Mgat3)_Wt Kv3.1b cell lines were more likely to express more than three neurites per cell than either NB_1_Wt Kv3.1b or NB_1(-Mgat2)_Wt Kv3.1b cell lines. Types of N-glycans altered the length of neurites in NB cells. Neurite length of both NB_1(-Mgat1) and NB_1(-Mgat2) cell lines were reduced to about 65% relative to those of NB_1 and NB_1(-Mgat3) cells (Figure 5C). When Wt Kv3.1b was expressed in both NB_1 and NB_1(-Mgat3) cells, there was a decrease in neurite length while expression in NB_1(-Mgat1) caused an increase in neurite length. The expression of Wt Kv3.1b in NB_1(-Mgat2) cells lacked an effect on neurite number and neurite length. Hence, NB cells expressing more processed N-glycans had longer neurites than those with less processed N-glycans. Further expression of Wt Kv3.1b in the various cell lines could impact neurite number and length.

NB cells were characterized by the percent of neurites that had structural projections from their tips, referred to as neurite tip projections (Figure 5D). In all cases, the non-transfected cell lines had similar levels of neurite tip projections. Expression of Wt Kv3.1b in NB_1(-Mgat1) had a significant increase in the number of neurite tip projections relative to its non-transfected cell line, as well as the other Kv3.1b expressing NB cell lines. To further elaborate, the neurite tip projections were categorized as having hair-like projections (filopodia), sheet-like projections (lamellipodia) and hair-like and sheet-like projections (filopodia/lamellipodia), respectively (Figure 5E–G). Non-transfected NB_1, NB_1(-Mgat1), and both NB_1(-Mgat2) and NB_1(-Mgat3) cell lines had the highest level of filopodia, lamellipodia and filopodia/ lamellipodia, respectively. Expression of Wt Kv3.1b in NB_1 cells greatly reduced and increased the levels of filopodia and lamellipodia, respectively. Kv3.1b expression in NB_1(-Mgat1) cells had decreases and increase in lamellipodia and filopodia/lamellipodia, respectively. The NB_1(-Mgat3)_Wt Kv3.1b cells had a decrease in the level of filopodia, like the parental cell line, but had an increases in the percent of filopodia/lamellipodia. The types of neurite tip projections were unchanged in NB_1(-Mgat2) when they were transfected with Wt Kv3.1b. Overall, all four non-transfected cell lines had different levels of filopodia, lamellipodia, and filopodia/lamellipodia at the tips of their neurites. The percent of neurite tip projections only increased in NB_1(-Mgat1) cells in response to expression of Wt Kv3.1b; however, the types of structures at the tips of the neurites were different for NB_1_Wt Kv3.1b, NB_1(-Mgat1)_Wt Kv3.1b and NB_1(-Mgat3)_Wt Kv3.1b cell lines compared to their non-transfected cell lines. Of note, the morphology of NB_1(-Mgat2) cells were unchanged by the expression of Wt Kv3.1b. These results demonstrate that N-glycan populations and Kv3.1b expression can modify NB cell morphology.

### Expression of glycosylated Kv3.1b enhances NB migratory rates

**Figure 6:**
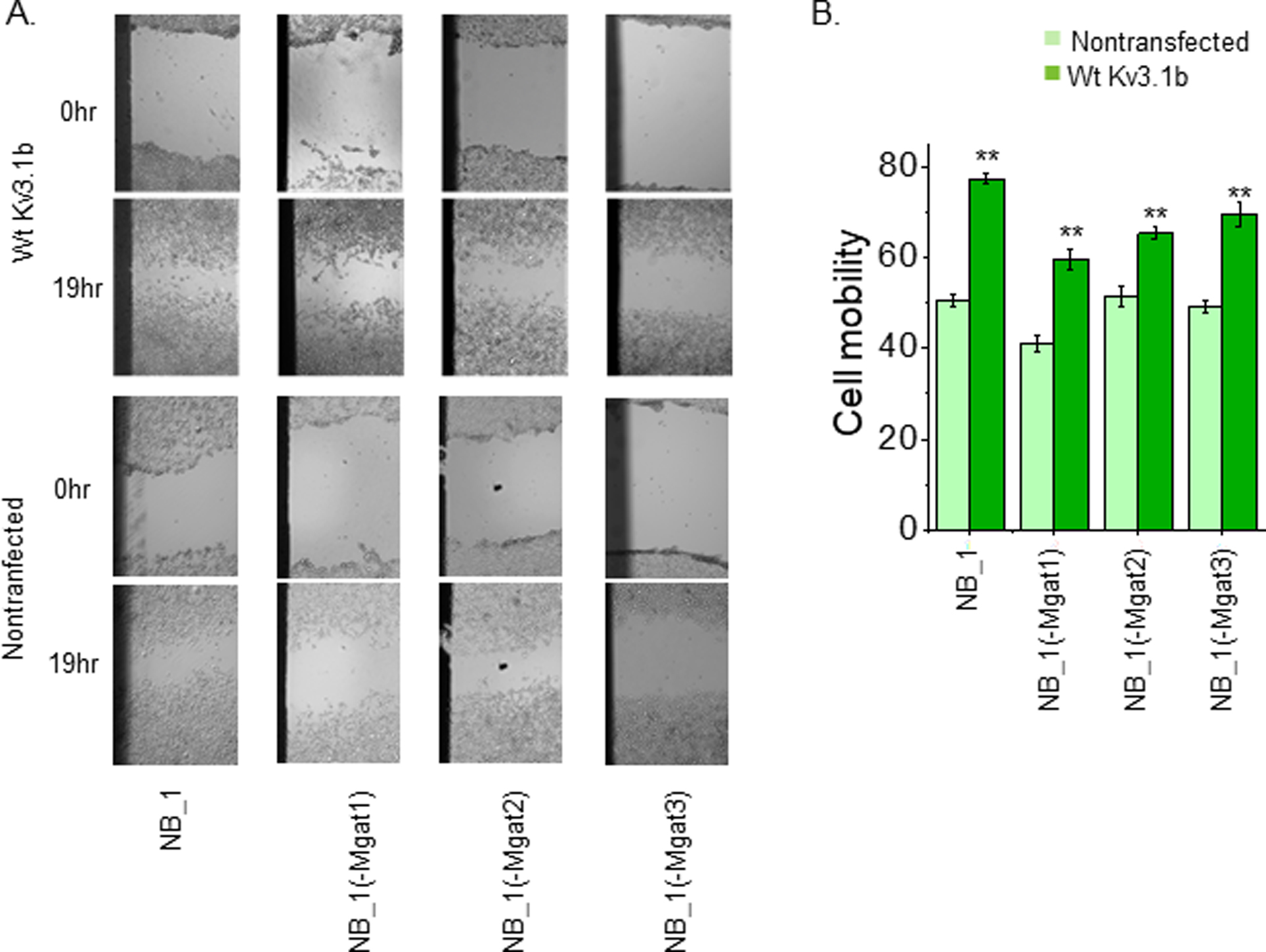
N-glycan type associated with the Kv3.1b glycoprotein influences NB migratory rates. Representative images of cell wounds at 0 hours and 19 hours in non-transfected NB_1, NB_1 (-Mgat1), NB_1 (-Mgat2) and NB_1 (-Mgat3) (lower panels) or glycosylated Kv3.1b transfected (upper panels) (A). Bar graph illustrates cell mobility based on percent wound closure at 19h (B). Data are presented as mean ± SEM (n≥6) and Wt. Kv3.1 expressing NB cell line was compared to non-transfected NB cell line by student t-test (**p< 0.001).

Cell migratory rates between parental and N-glycosylation mutant cell lines had less than 10% differences [30,31,33,34]. Here each of the non-transfected cell lines was compared to their respective cell line expressing Wt Kv3.1b. Cell wounds were produced in NB_1, NB_1 (-Mgat1), NB_1 (-Mgat2) and NB_1 (-Mgat3) cells expressing Wt Kv3.1b and then allowed to recover for 19h (Figure 6A, upper panels). The non-transfected cell lines were conducted in parallel (Figure 6A, lower panels). The percent of wound closures were determined to quantify the cell mobility of each cell line (Figure 6B). In all cases, expression of Wt Kv3.1b increased cell migratory rates relative to their respective non-transfected cell line. Moreover, expression of Wt Kv3.1b in NB_1 and NB_1(-Mgat1) cells had larger changes in cell mobility compared to the other two cell lines (NB_1, 0.27±0.02, n=22; NB_1(-Mgat1), 0.21±0.05, n=13); NB_1(-Mgat2), 0.07± 0.02, n=39); and NB_1(-Mgat3), 0.11±0.04, n=6). Thus, these results indicate that expression of glycosylated Kv3.1b enhances NB cell migratory rates.

### Impact of N-glycans and Kv3.1b on actin polymerization/depolymerization and intermediate filaments in NB cells

**Figure 7:**
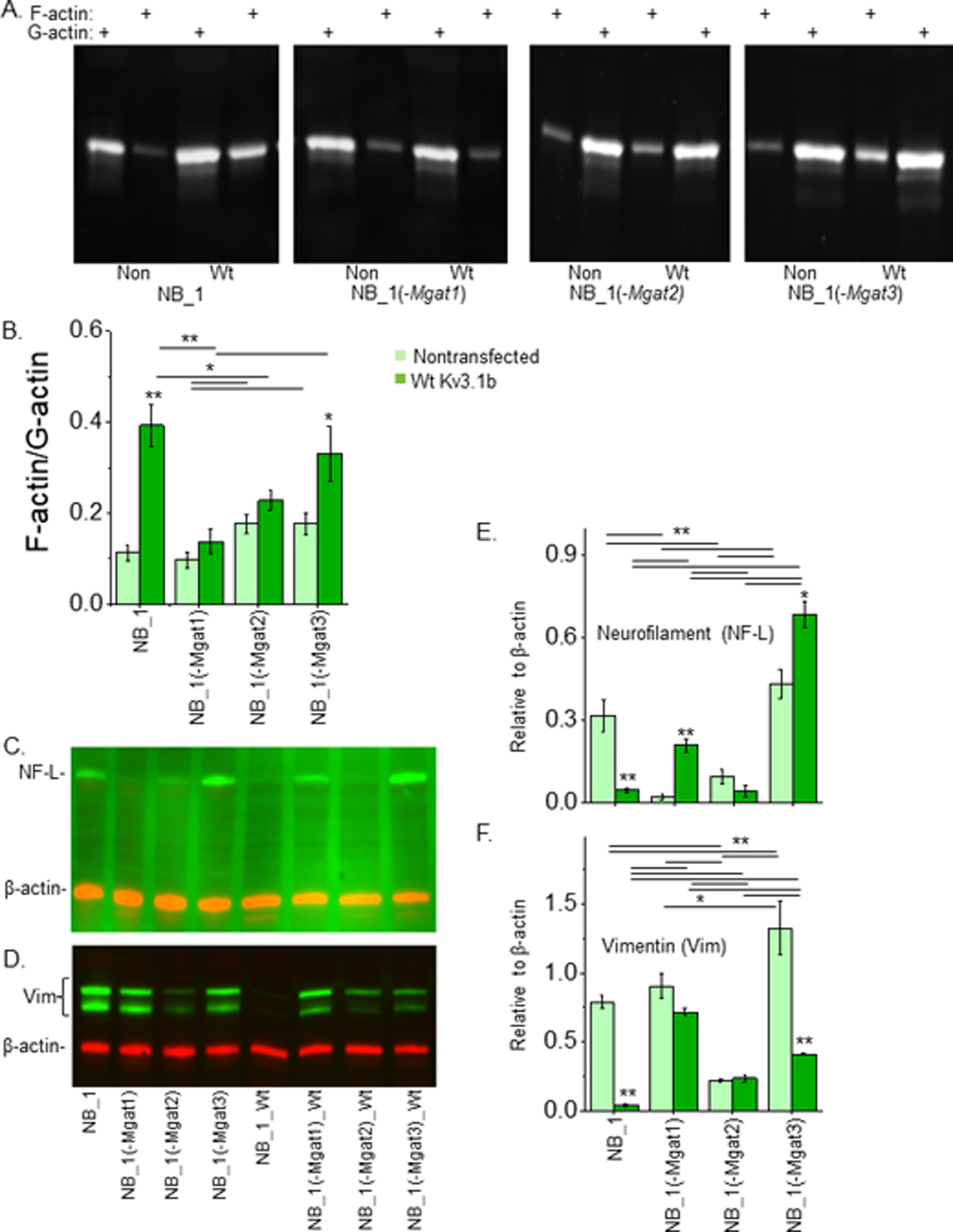
Influence of N-glycans and Kv3.1b on actin polymerization and intermediate in NB cells. Western blots (n≥3) of filamentous and globular actin (A) and quantification (B) in NB_1 (n=5,4), NB_1 (-Mgat1) (n=5, 4), NB_1 (-Mgat2) (n=4, 3), and NB_1 (-Mgat3) (n=4, 3) cells without Kv3.1 (light green) or expressing Kv3.1 WT (dark green). Western blots of Neurofilament-L (NFL; n=4) (C) and Vimentin (Vim; n=3) (D) multiplexed with β-actin: rhodamine in cell lines as indicated. Quantification of protein levels relative to β-actin (E, F) in NB_1, NB_1 (-Mgat1), NB_1 (-Mgat2), and NB_1 (-Mgat3) cells without Kv3.1 (light green) or expressing Kv3.1 WT (dark green). Data are presented as mean ± SEM. Wt Kv3.1 expressing NB cell line was compared to non-transfected NB_1 cell line by student t-test (*p < 0.05, **p < 0.001) and non-transfected and Wt Kv3.1b expressing NB_1 cell line were all compared by one- way ANOVA with Holm-Bonferroni mean comparison (*p < 0.1, **p < 0.05).

Since cell morphology and migration were changed by N-glycan populations and Kv3.1b expression, it was determined whether these changes in the cells were accompanied by differences in the cytoskeleton. In all cases, the level of F-actin was lower than G-actin (Figure 7A). Quantification of F-actin to G-actin levels were lowest in NB_1(-Mgat1), along with NB_1, while highest in NB_1(-Mgat2) and NB_1(-Mgat3) (Figure 7B). The amount of F-actin dramatically increased when Wt Kv3.1b was expressed in NB_1 and NB_1(-Mgat3) cells. In terms of intermediate filaments, neurofilament-light chain (NF-L) immuno-bands were of high intensity in NB_1, NB_1(-Mgat3), NB_1(-Mgat1)_Wt Kv3.1b, and NB_1(-Mgat3)_Wt Kv3.1b cell lines (Figure 7C) while immuno-bands of vimentin (Vim) were of strong intensities in NB_1, NB_1(-Mgat1), NB_1(-Mgat3), and NB_1(-Mgat1)_Wt Kv3.1b, and those of intermediate intensities were in NB_1(-Mgat2), NB_1(-Mgat2)_Wt Kv3.1b, and NB_1(-Mgat3)_Wt Kv3.1b (Figure 7D).

Quantification of the NF-L immunobands in the nontransfected cell lines showed highest expression in NB_1(-Mgat3) and NB_1 cell lines while the levels in NB_1(-Mgat1) and NB_1(-Mgat2) cell lines were much lower (Figure 7E). Expression of Wt Kv3.1b in NB_1 decreased NF-L levels while it increased levels in NB_1(-Mgat1) and NB_1(-Mgat3) cells. Vimentin abundance was lowest in NB_1(-Mgat2) cells, intermediate in NB_1 and NB_1(-Mgat1), and highest in NB_1(-Mgat3) cells (Figure 7F). Expression of Wt Kv3.1b in NB_1 and NB_1(-Mgat3) cells greatly reduced vimentin levels while vimentin levels were unchanged in NB_1(-Mgat1) and NB_1(-Mgat2) cells expressing Wt Kv3.1b. Taken together, the data indicated that the cytoskeletal structure was different in all four cell lines due changes in N-glycan structures. Moreover, Kv3.1b expression in NB_1, NB_1(-Mgat1), and NB_1(-Mgat3) cells impacted expression of F-actin, neurofilament and vimentin while Kv3.1b expression in NB_1(-Mgat2) cells had no effect on the expression of the cytoskeletal proteins.

### Relationship of N-glycans on spatial arrangement of glycosylated and unglycosylated Kv3.1 proteins in membranes of NB cells

**Figure 8:**
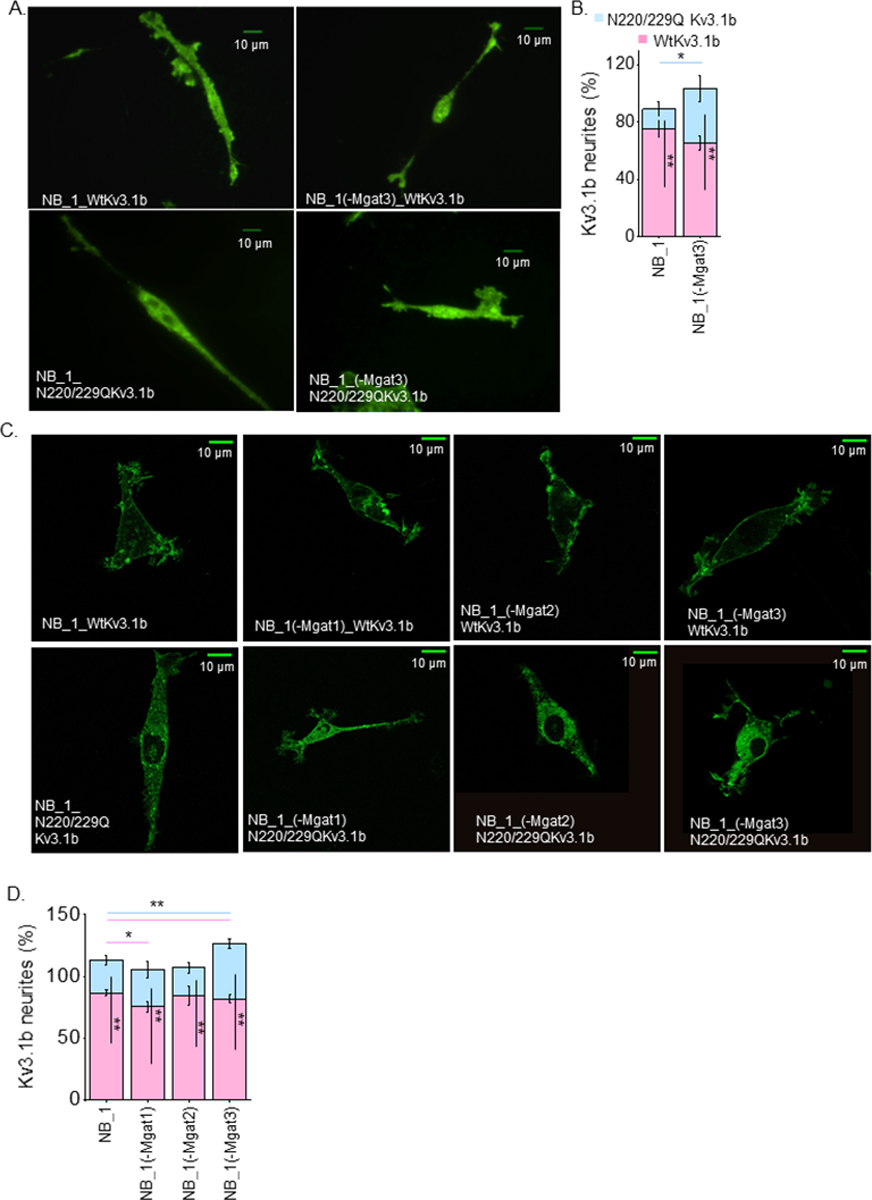
Kv3.1b localization in outgrowths of rat neuroblastoma cell lines with atypical N-glycosylation. Representative TIRF (A) microscopy images acquired of glycosylated (WT) (upper panels) and glycosylated (N220/229Q) (lower panels) Kv3.1b proteins labeled with EGFP expressed in NB_1 (left columns) and NB_1(-Mgat3) (right columns) cells. Quantification of percent of Wt Kv3.1b (pink) and N220/229Q Kv3.1b (blue) localized in neurites of NB_1 and NB1_(-Mgat3) cells as detected via TIRF (n≥18) (B). Confocal microscopy images (C) of glycosylated (WT) (upper panels) and unglycosylated (N220/229Q) (lower panels) Kv3.1b proteins. in cell lines as indicated. Bar graphs indicate % Kv3.1 detected in neurites via confocal microscopy (n≥10) (D) in NB cell lines as shown. The Wt Kv3.1 expressing NB cell line was compared to N220/229Q Kv3.1b expressing NB cell line by student t-test (*p < 0.05, **p < 0.001). Wt Kv3.1b or N220/229Q Kv3.1b expressing NB cell lines were compared by one-way ANOVA with Holm-Bonferroni mean comparison (*p<0.1, **p<0.05). Black lines inside the bars denote statistical differences between WT_Kv3.1b and N220/9Q Kv3.1b within each cell line. Corresponding color lines above the bar graph indicate statistical differences among the cell lines.

It was shown that the distribution of glycosylated Kv3.1b to the neurites was higher than the unglycosylated Kv3.1b protein in NB_1 cells and primary cortical/hippocampal neurons using TIRF microscopy [12] which measured the amount of Kv3.1b protein in or near the adhered plasma membrane. Micrographs using TIRF microscopy were captured for control cells (NB_1_Wt and NB_1_N220/229Q) and the NB_1(-Mgat3) cell lines stably expressing Wt Kv3.1b or N220/229Q Kv3.1b (Figure 8A). In all cases, glycosylated and unglycosylated Kv3.1b proteins were distributed between the soma and neurites. Quantification showed that more glycosylated Kv3.1b in NB_1(-Mgat3) went to the neurites than its unglycosylated counterpart, like the control (Figure 8B). However, the level of unglycosylated Kv3.1b in neurites of NB_1(-Mgat3) cells were greater than that of the control (NB_1 cell line). To extend our findings using a different technique, total Kv3.1b protein in cell membranes of neurites and cell body was measured by employment of confocal microscopy. Confocal micrographs of the parental and N-glycosylation mutant cell lines expressing either Wt or N220/229Q Kv3.1b support that more glycosylated Kv3.1b protein was trafficked to the neurites than the unglycosylated protein (Figure 8C). Further this finding is supported by quantification of Kv3.1 in neurites and soma in parental and N-glycosylation mutant cell lines expressing either Wt or N220/229Q Kv3.1b (Figure 8D). There was also a significantly higher amount of N220/229Q Kv3.1b in neurites for NB_1(-Mgat3) compared to NB_1_ N220/229Q Kv3.1b cells. Both TIRF and confocal microscopy measurements indicated that more of the unglycosylated Kv3.1b protein expressed in NB_1(-Mgat3) cells was in neurites than soma relative to the parental cell line. These results support that the N-glycan attached to the Kv3.1b protein enhanced its distribution to neurites relative to the soma. Further this distribution was similar if determined for Kv3.1b in adhered membrane or both adhered and non-adhered membranes of cells.

### Expression levels of ER folding and transport proteins in various NB cells expressing glycosylated and unglycosylated Kv3.1 proteins

**Figure 9:**
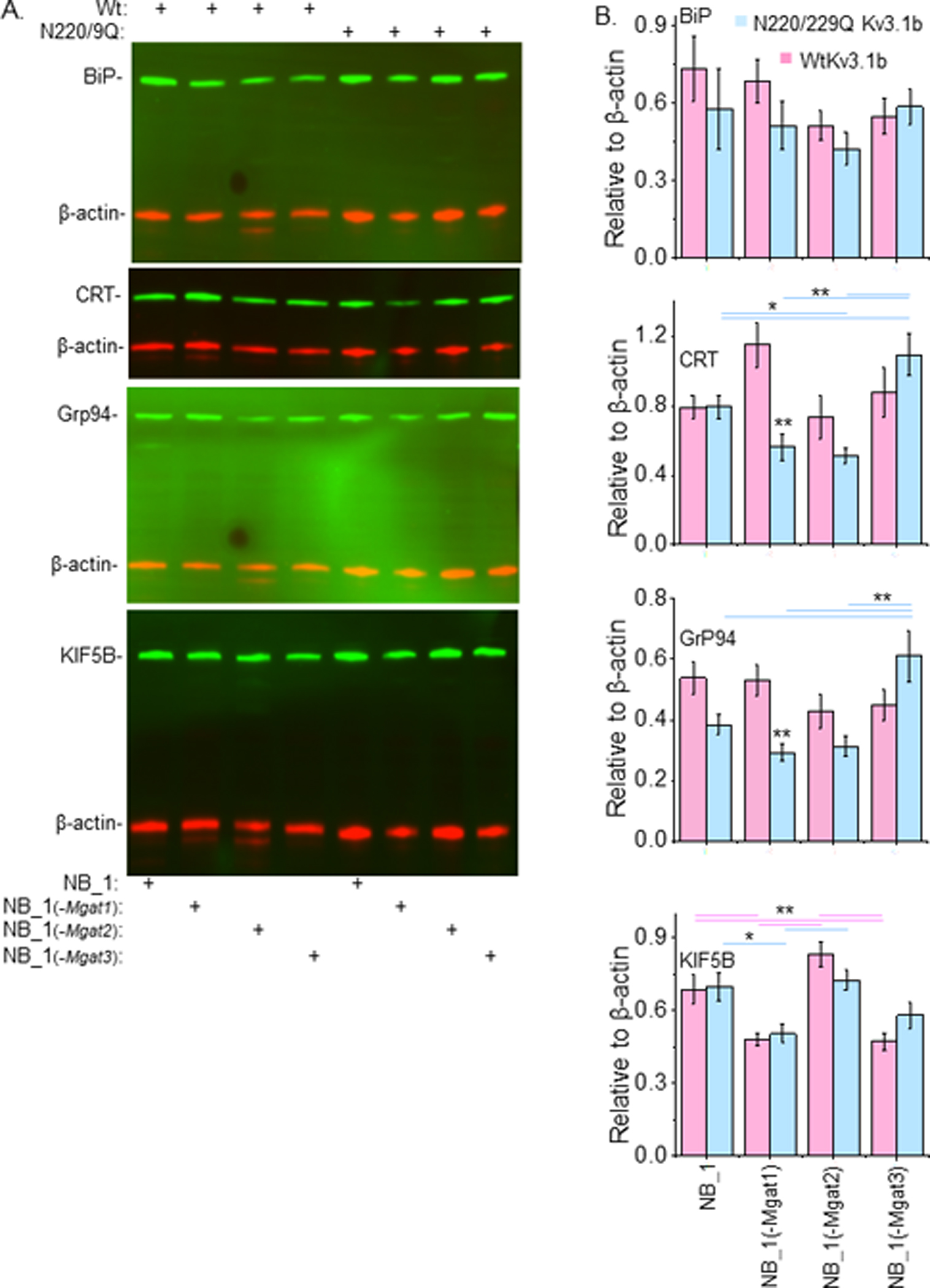
N-glycans and expression of ER chaperones in neuronal cells. Western blots of BiP (n=5), calreticulin (CRT) (n=4), Grp94 (n=4), and KIF5B (n=4) multiplexed with β-actin: rhodamine (A) and their quantification in NB cells (as shown) expressing WT KV3.1 (pink) or N220/9Q_Kv3.1 (blue)(B). Corresponding color lines above the bar graph indicate statistical differences among the cell lines. (*, ***) inset within the bar graph signified differences between WT_Kv3.1b and N220/9Q Kv3.1b within indicated cell line. Data are presented as mean±SEM. Wt. Kv3.1 expressing NB cell line was compared to non-transfected NB_1 cell line by student t-test (*p<0.05, **p<0.001). Wt. Kv3.1b or N220/9Q Kv3.1b expressing NB cell lines were all compared by one-way ANOVA with Holm-Bonferroni mean comparison (*p < 0.1, **p < 0.05).

Here the aim was to determine whether unglycosylated Kv3.1b proteins in NB cells with different N-glycosylation pathways had greater difficulties in folding and ER exit than glycosylated Kv3.1b protein by evaluating levels of ER chaperone and transport proteins. The three chaperone proteins evaluated were BiP, Grp94, and Calreticulin (CRT). The two earlier chaperones bind to exposed protein regions of unfolded proteins while the later one binds to specific N-glycan structures of nascent glycoproteins. The ER-to-Golgi transport protein is KIF5B. Representative Western blots of BiP, CRT, GRP94 and KIF5B (Figure 9A), and mean intensities of a given immunoband relative β-actin were plotted to bar graphs (Figure 9B). In several cases, the loads were inconsistent between samples; however, using multiplex for western blotting corrects for this as the ratio of protein of interest to β-actin for a single sample are averaged and reported as relative to β-actin. As such, when evaluating immunoband intensities it is more accurate to compare immunoband of interest to that of the β-actin in each sample. The levels of BiP were not significantly different for parental and N-glycosylation mutant cell lines expressing Wt or N220/229Q Kv3.1b proteins. Expression levels of CRT were higher in NB_1(-Mgat1) cells expressing of Wt Kv3.1b relative to those expressing N220/220Q Kv3.1b. When comparing the various cell lines expressing unglycosylated Kv3.1b a significant increase was detected in NB_1(-Mgat3) cells. Levels of GRP94 were higher in NB_1(-Mgat1) cells expressing of Wt Kv3.1b relative to those expressing N220/220Q Kv3.1b. Further NB_1(-Mgat3)_N220/229Q Kv3.1b had higher levels of GRP94 than the other three cell lines expressing unglycosylated Kv3.1b. The levels of KIF5B were similar between each of the respective cell lines expressing Wt or N220/229Q Kv3.1b proteins. However, both NB_1_WtKv3.1b and NB_1(-Mgat2)_WtKv3.1b cell lines expressed more KIF5B than NB_1(-Mgat1)_WtKv3.1b and NB_1(-Mgat3)_WtKv3.1b. Thus, these results indicate that expression of the unglycosylated Kv3.1b protein could fold to its mature form and exit the ER with similar ease as the glycosylated Kv3.1b protein since the levels of ER folding and trafficking proteins were not increased in NB cell lines expressing unglycosylated Kv3.1b.

## Discussion

Our current study showed that expression of Kv3.1 in NB cells with different perturbations in their N-glycosylation pathway resulted in changes in their N-glycan populations. The NB_1, NB_1(-Mgat1), NB_1(-Mgat2), and NB_1(-Mgat3) cell lines had N-glycan populations like those identified by employment of ESI-MS [30]. When NB cells were stably transfected with glycosylated (Wt) Kv3.1b, the level of complex type N-glycans were increased in NB_1, NB_1(-Mgat2), and NB_1(-Mgat3) cell lines. Moreover, the percent of triantennary were raised from 2 to 6.7-fold in the various Kv3.1b-expressing cells. Increases in hybrid type N-glycans were also observed in cell lines with an intact Mgat2 (NB_1, and NB_1(-Mgat3)). In addition to N-glycans with more branchpoints, there was more fucosylated complex and hybrid types of N-glycans in the various cell lines. These results, along with the slower electrophoretic mobility Kv3.1b expressed in NB_1(-Mgat3), support that the N-glycans of Kv3.1b are more elaborate. It should also be mentioned that decreased activity of GnT-II and -III in the various cell lines do not appear to reduce activity of glycosyltransferases (e.g., GnT-II, -III, and -IV, ST8Sia-II and -IV) that undergo N-glycosylation processing [35] since Kv3.1b expression caused similar trends in N-glycans of the various NB cell lines. Further changes in cell migration were increased in Kv3.1b-expressing NB cells, suggesting that neither poly-α-2,8-sialyltransferases, ST8SiaII and ST8SiaIV, had reduced activity via neural cell adhesion molecule (NCAM) [36]. Hence, the expression of Kv3.1b in NB cells promote the synthesis of more elaborate N-glycans via addition of antenna and fucose residues.

The different N-glycan populations were accompanied with modifications in neurite structure. We observed that neurites were longer in NB_1 and NB_1(-Mgat3) cell lines than NB_1(-Mgat1) and NB_1(-Mgat2) cell lines, and that neurite tip projections were different in all cell lines. These results support changes in N-glycosylation processing of proteins can alter cell morphology. Expression of glycosylated Kv3.1b in NB_1(-Mgat1) and NB_1(-Mgat3) cells increased neurite number, while neurite length was decreased and increased in both NB_1 and NB_1(-Mgat3) and NB_1(-Mgat1) cells, respectively, expressing Kv3.1b. The percent of neurite tips for NB_1(-Mgat1)_Wt Kv3.1b cells were raised while the other NB cell lines lacked this change. Further the type of structures formed at the tip of the projections were modified for NB_1_Wt Kv3.1b, NB_1(-Mgat1)_Wt Kv3.1b, and NB_1(-Mgat3)_Wt Kv3.1b relative to their nontransfected counterparts. NB_1(-Mgat2) cells expressing Wt Kv3.1b were virtually identical to nontransfected NB_1(-Mgat2). These changes in neurite structure were supported by different levels of cytoskeletal proteins, including F-actin, neurofilament and vimentin. In short, the cytoskeletal networks were different in the various NB cell lines and furthermore expression of glycosylated Kv3.1b in all NB cell lines, except NB_1(-Mgat2), had different cytoskeletal protein expression patterns. Taken together, these results support that change in N-glycan populations and glycosylated Kv3.1b expression alter the cytoskeletal network in the various NB cell lines, and consequently cell morphology. An anomaly was the expression of Kv3.1b in NB_1(-Mgat2) cells which lacked changes in neurite structure and cytoskeletal network. The reason for this could be due the attachment of hybrid N-glycans to glycosylated Kv3.1b [12], and/or the high levels of hybrid N-glycans (>30%) in NB_1 cells with a mutated Mgat2 gene. Clearly, more future research is warranted to address the role hybrid N-glycans in making the NB cells resistant to changes in their cytoskeleton, as well as cell morphology, when Kv3.1b is expressed in the cell line.

The changes in the cytoskeletal network could explain the different NB cell morphologies. It is well-established that lamellipodia structures are rich in actin while filopodia are composed of microtubules [37]. Here a significant increase in F-actin was observed when the percent of filopodia was decreased and the lamellipodia containing structures were increased in Kv3.1b-expressing NB_1 and NB_1(-Mgat3) cells. Regarding NB_1(-Mgat1) and NB_1(-Mgat2) cell lines, their levels of F-actin were unchanged as was ratio of filopodia to lamellipodia without and with Kv3.1b expression. The levels of neurofilament were greatly decreased and increased in NB_1_Wt Kv3.1b and NB_1(-Mgat1)_Wt Kv3.1b cells, respectively, compared to their nontransfected counterparts, which correlated with decreased and increased neurite length. These results agree with past structural studies that showed axons are composed of combined neurofilaments and microtubules [37–39]. In all cases, we found that expression of Kv3.1b in the NB cell lines enhanced cell migration. However, there was no correlation with the abundancies of the cytoskeletal proteins, such as vimentin, which has been shown to increase cell migration [40].

Our past studies established a mechanism of N-glycosylation processing of Kv3.1b in modulating its spatial arrangement and Kv3 channel activity in NB cells and primary neurons [12], and furthermore we showed that fast-spiking motor neurons were maldeveloped in a multicellular organism, zebrafish, when Kv3.1b was not fully N-glycosylated [13]. A possible explanation for the hampered development could be due to ER function and/or its distribution between soma and neurites since a report showed that the N220Q Kv3.1b mutant protein was retained in the ER membrane of COS-7 cells [41]. This was addressed by expressing glycosylated and unglycosylated Kv3.1b in NB cells with different N-glycan populations. Our current results indicated that the glycosylated and unglycosylated Kv3.1b proteins were properly folding and being transported out of the ER, along with our past results that unglycosylated and partially glycosylated Kv3.1b proteins produced functional Kv3 channels in the plasma membrane [11]. However, the level of Kv3.1b in neurites was considerably reduced for the unglycosylated Kv3.1b protein compared to the glycosylated Kv3.1b protein. In comparing the distribution of glycosylated Kv3.1b in the various cell lines, less Kv3.1b was detected in neurites of NB_1(-Mgat1) and NB_1(-Mgat3) cells relative to NB_1 and NB_1(-Mgat2) cells, and furthermore levels of KIF5B, an axonal transport protein [20], were lower in NB_1(-Mgat1) and NB_1(-Mgat3) cells. Future studies should unravel the link between KIF5B and N-glycan populations in axonal transport of Kv3.1b. Thus, this comprehensive study of expressing Kv3.1b in neuronal-derived cell lines with different N-glycan populations support that transport of the Kv3.1b to neurites likely contributes more greatly to underdeveloped fast-spiking motor neurons in zebrafish than ER folding and transport proteins.

In conclusion, we employed a systematic approach to demonstrate that disruptions in the N-glycosylation pathway influences cellular properties in neuronal-derived cell models. Moreover, expression of an N-glycosylated Kv3.1b protein yielded further changes in the N-glycan population within each cell line, which is of great interest given the vital role of glycosylation on both the conducting [11–13] and non-conducting [7,11] functions of the Kv3 channel. Further, observations of neurite structure, including neurite tip projections, were associated with the levels of cytoskeletal proteins. Through the development of N-glycosylation mutant cell lines without and with ecotopic expression of glycosylated Kv3.1b, along with the assignment of N-glycan populations of these cell lines, we demonstrate the close association of N-glycan content and cytoskeletal network in neuronal-derived cell structure/function. Consequently, the potential for targeted glycotherapeutics for a variety of pathologies is enhanced from the results herein.

## Supplementary Material

WB Images&Analysis

supp figures/tables
