# Supplementary material for: Kv3-Expressing Cells Present More Elaborate N-Glycans with Changes in Cytoskeletal Proteins, Neurite Structure and Cell Migration
Source: COJ Biomed Sci Res. Author manuscript; Available in PMC 2024 Dec 30. (PMC11684427)
Supplement: WB Images&Analysis [file NIHMS2040166-supplement-WB_Images_Analysis.pdf]

Neurofilament

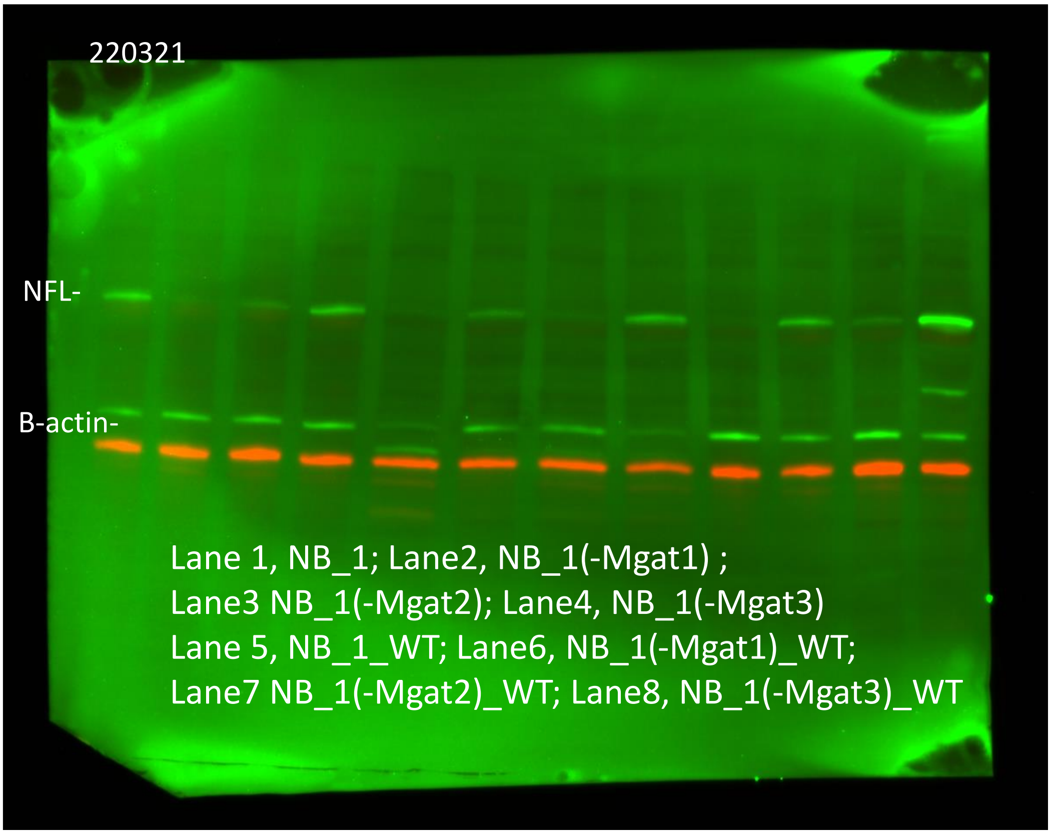

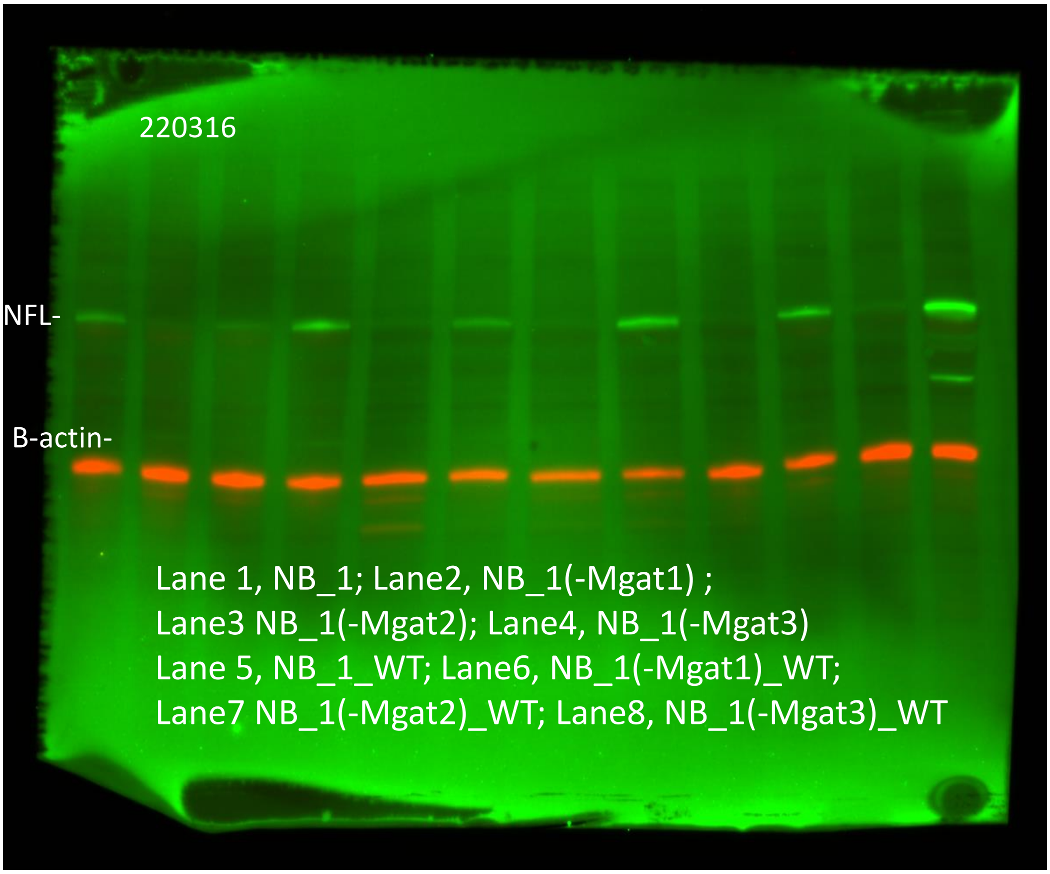

WT is WT Kv3.1b

Neurofilament

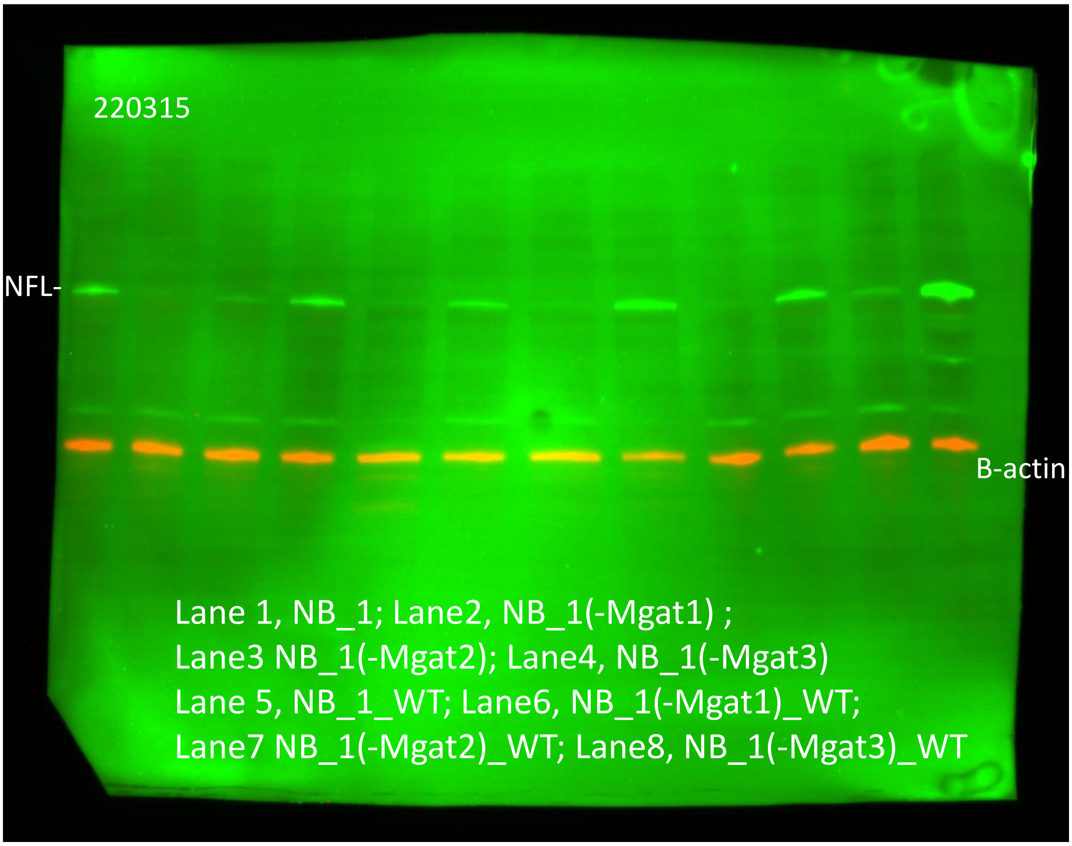

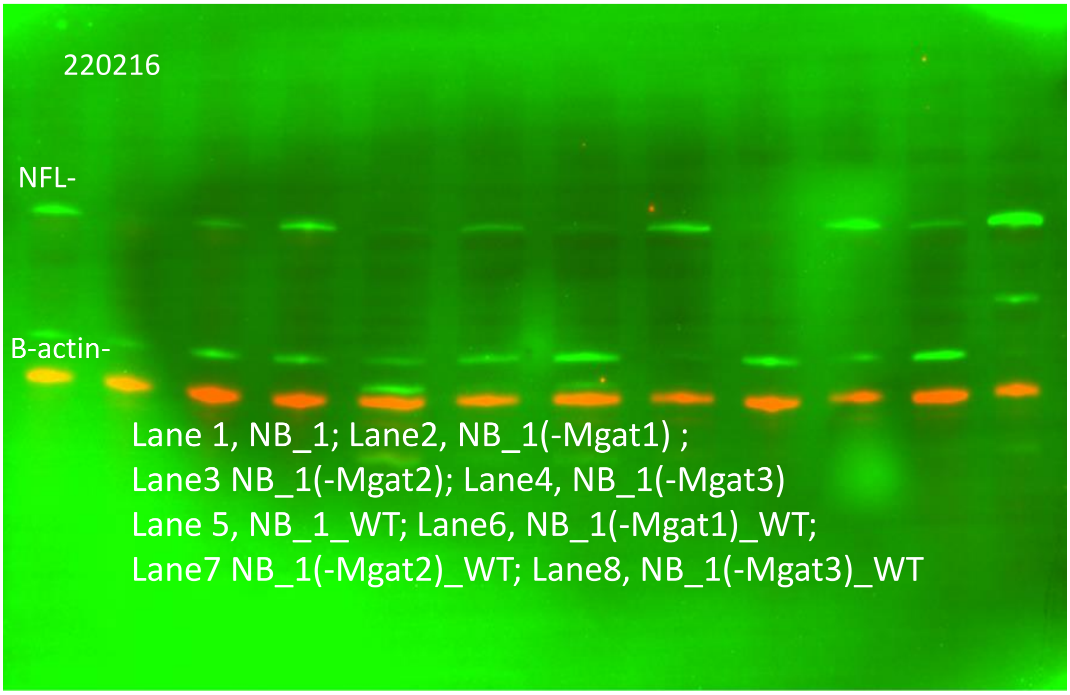

WT is WT Kv3.1b

Vimentin

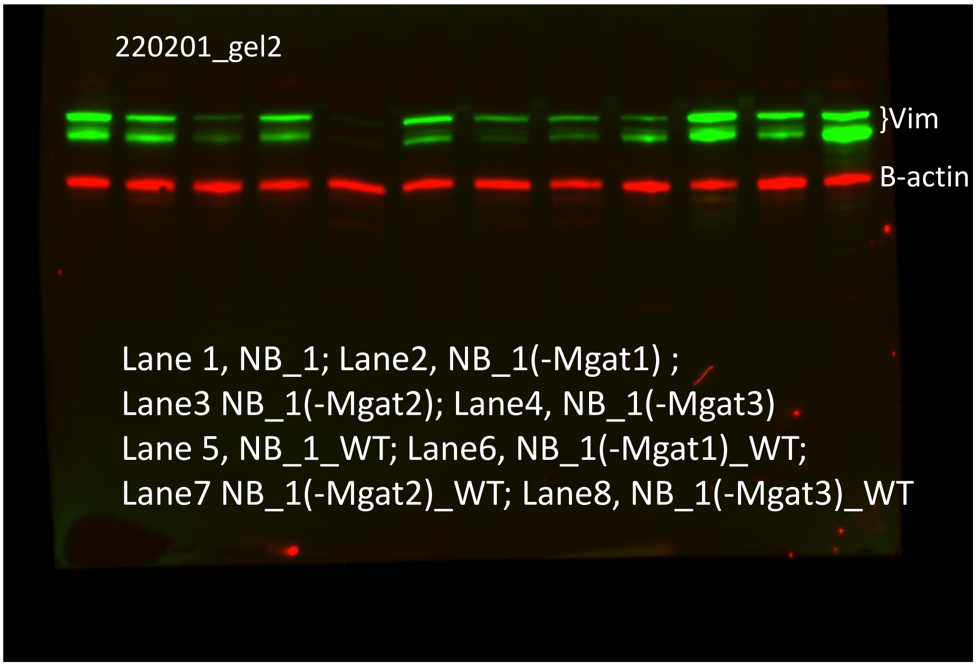

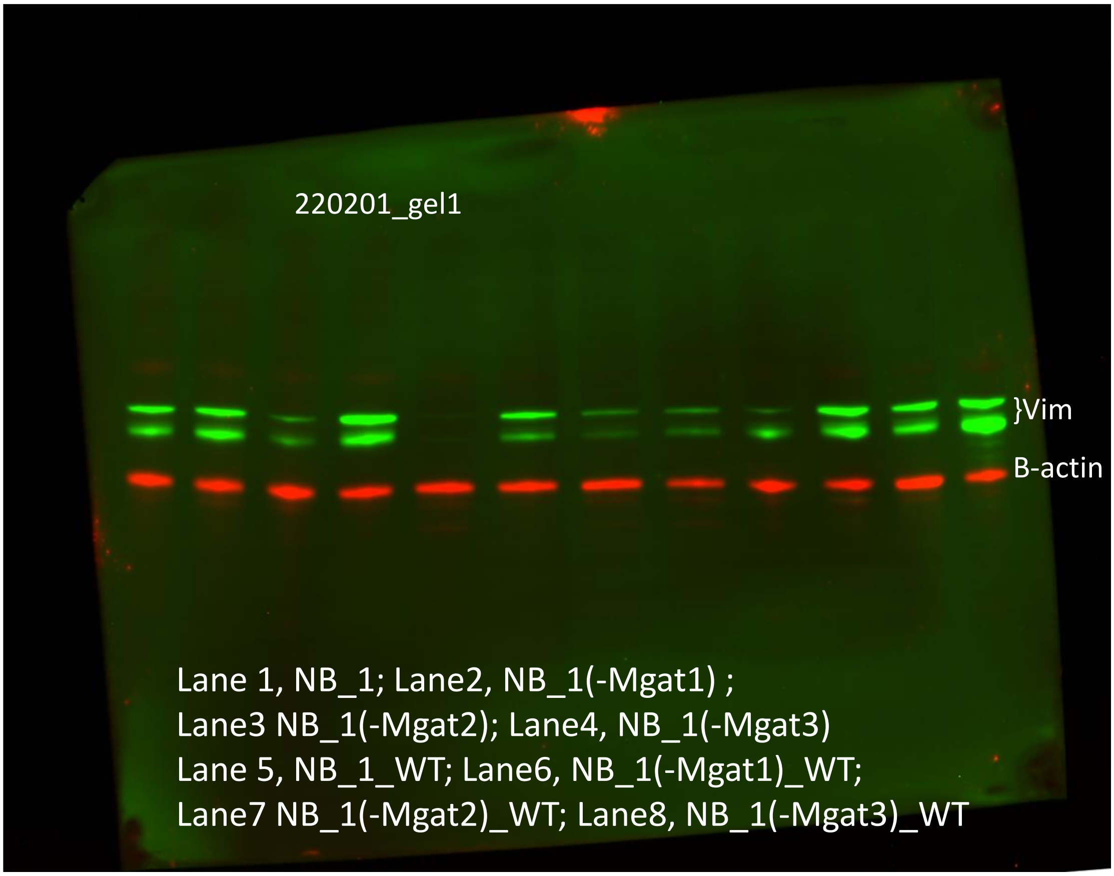

WT is WT Kv3.1b

Vimentin

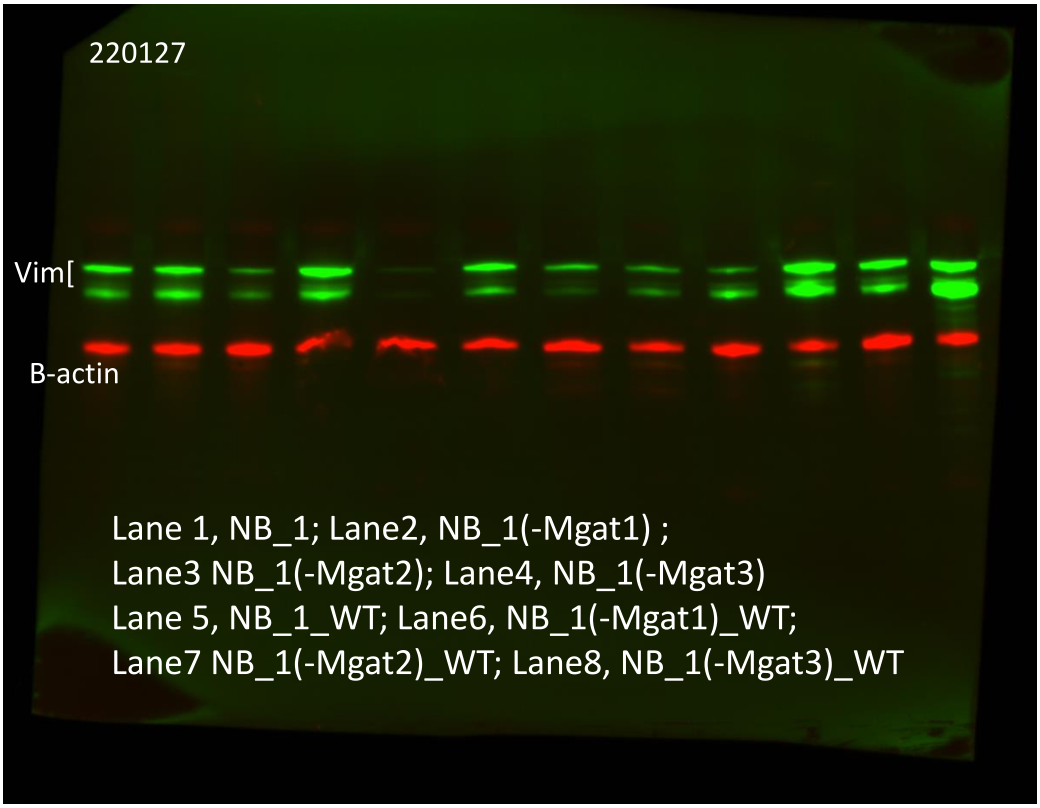

WT is WT Kv3.1b

KIF5B

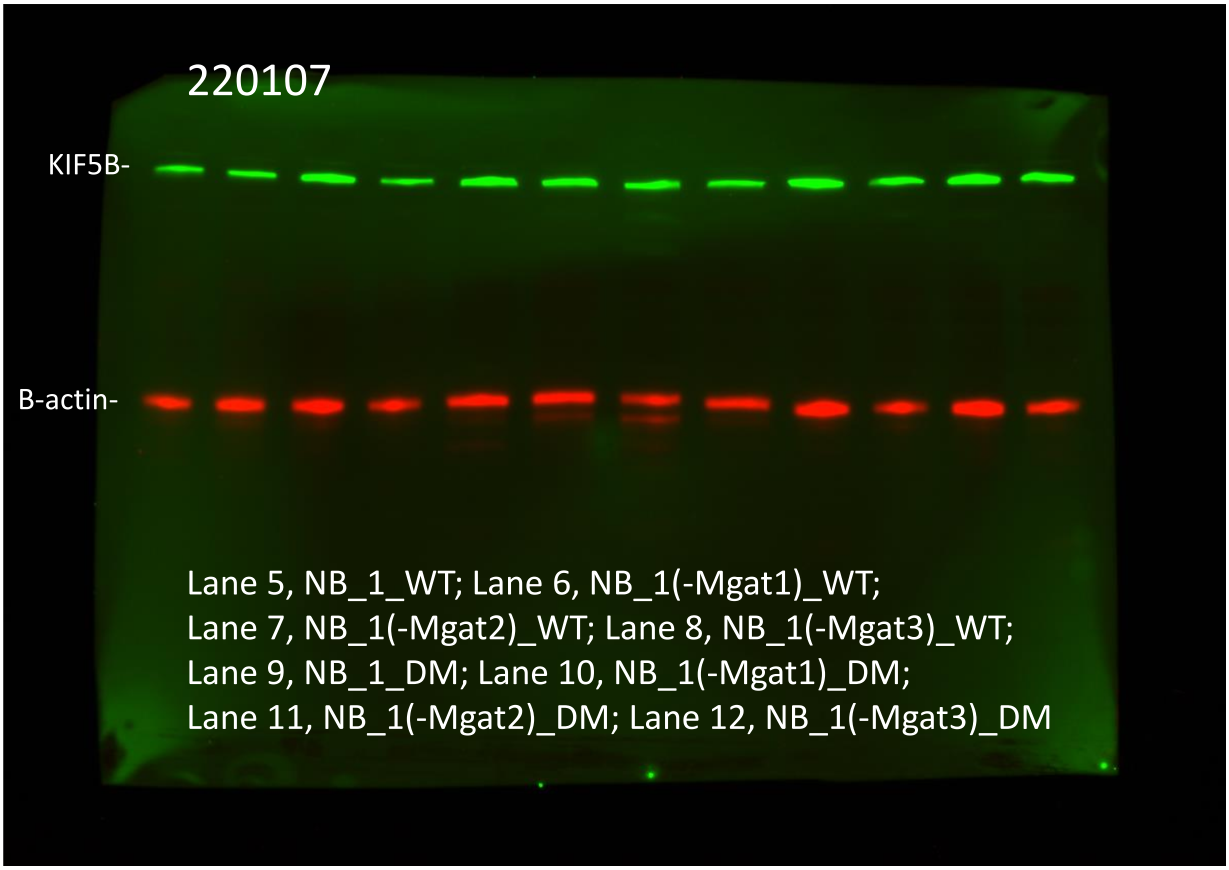

WT is WT Kv3.1b and DM is N220/229Q Kv3.1b

KIF5B

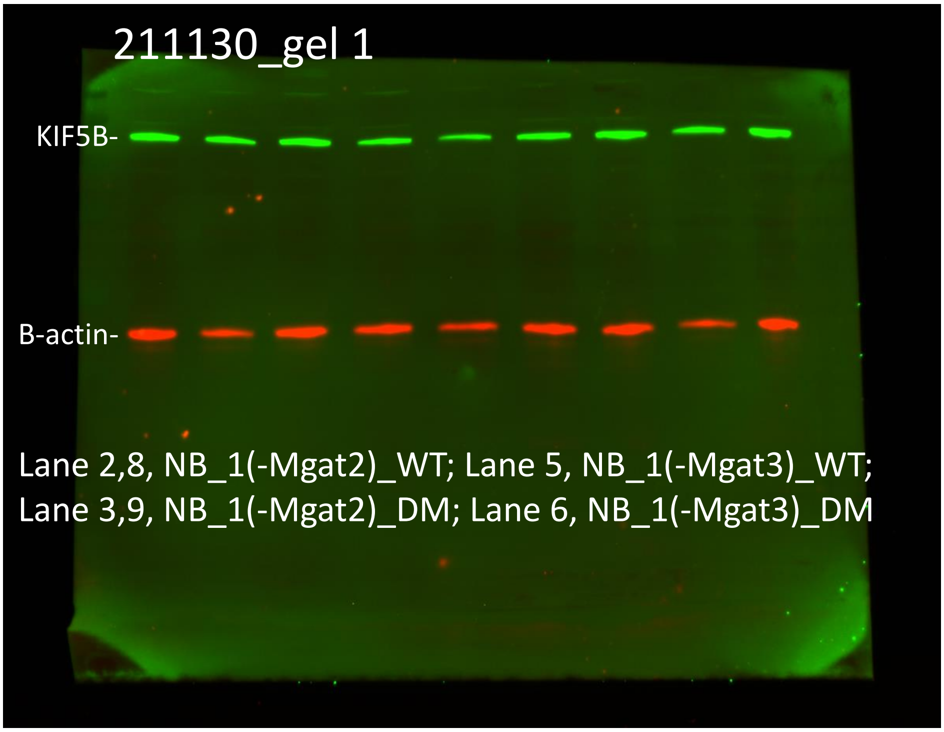

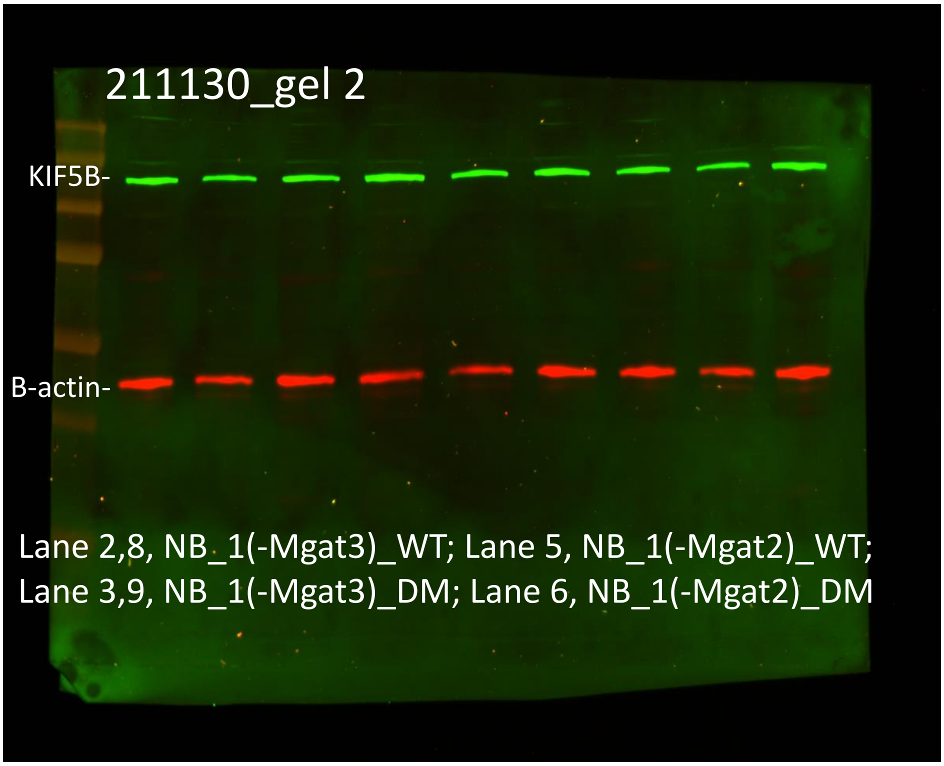

WT is WT Kv3.1b and DM is N220/229Q Kv3.1b

KIF5B

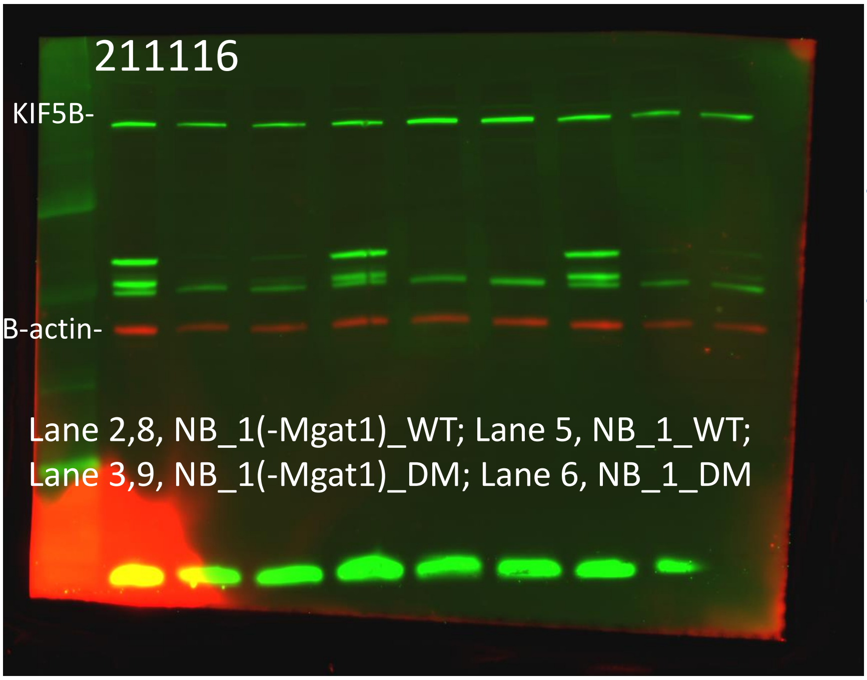

Multiplexing with other Antibodies, not background bands, see slide 6

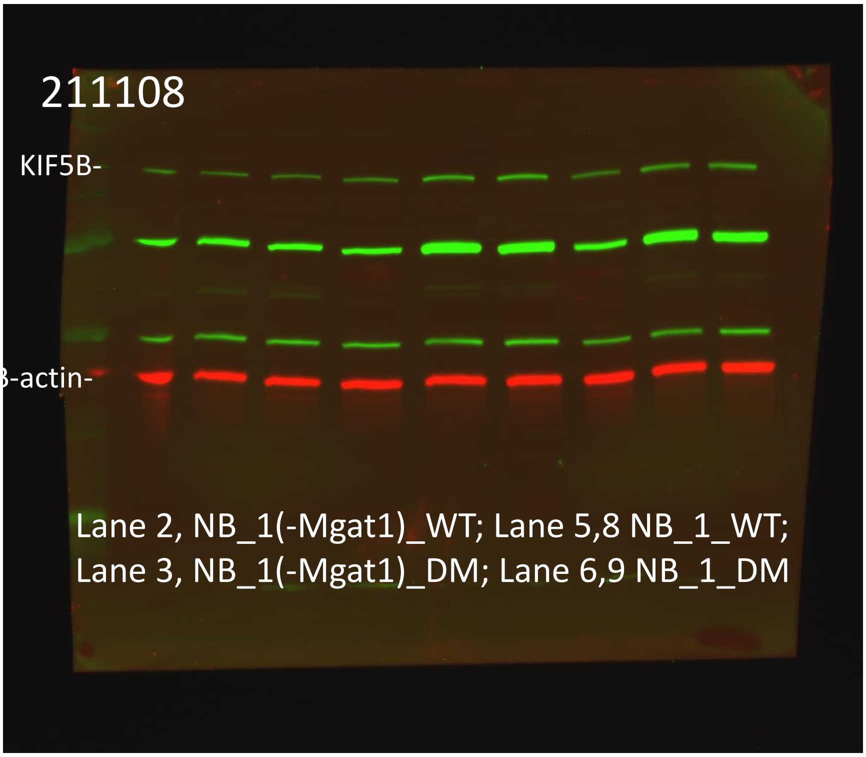

Multiplexing with other Antibodies, not background bands, see slide 6

WT is WT Kv3.1b and DM is N220/229Q Kv3.1b

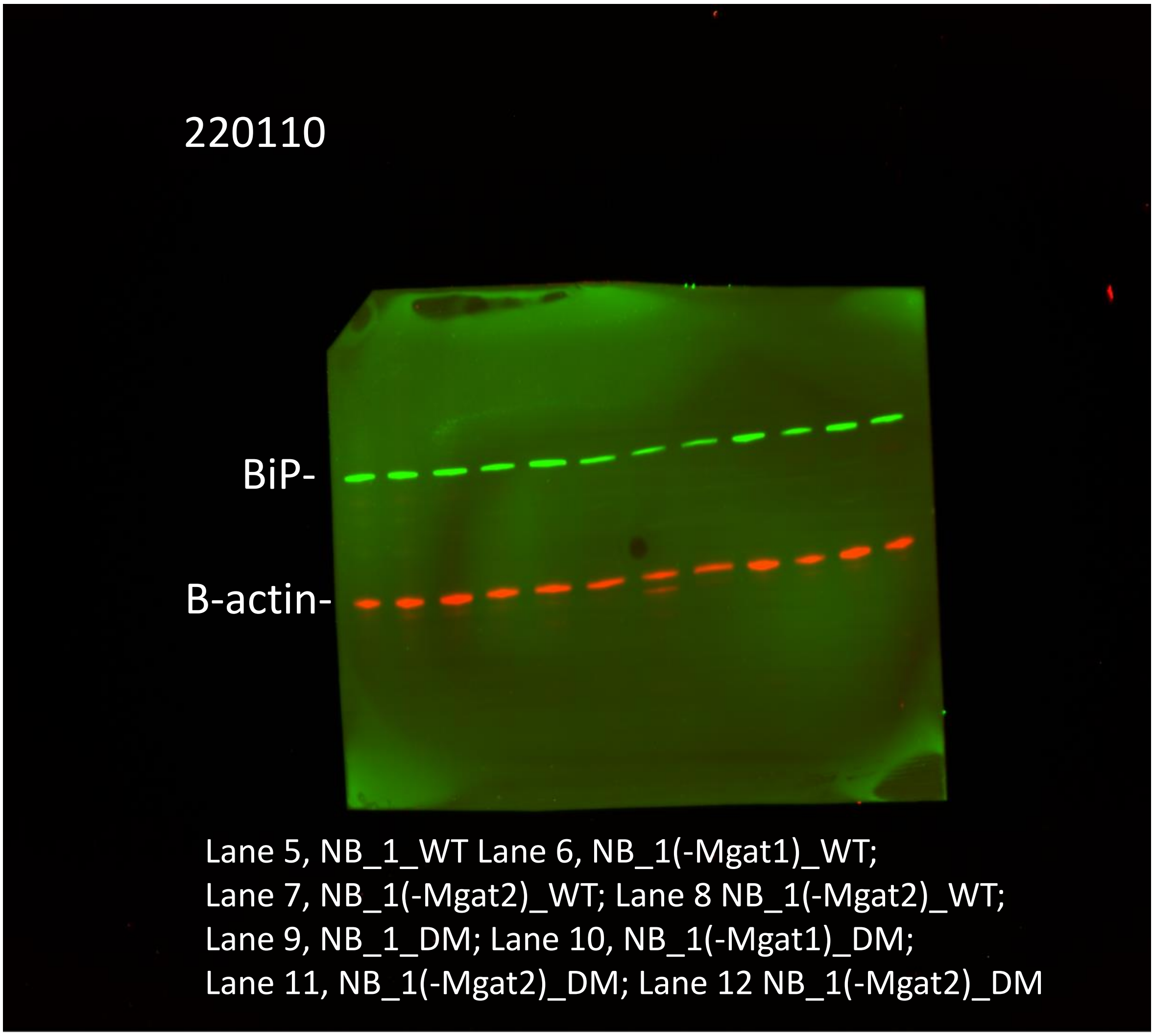

220110

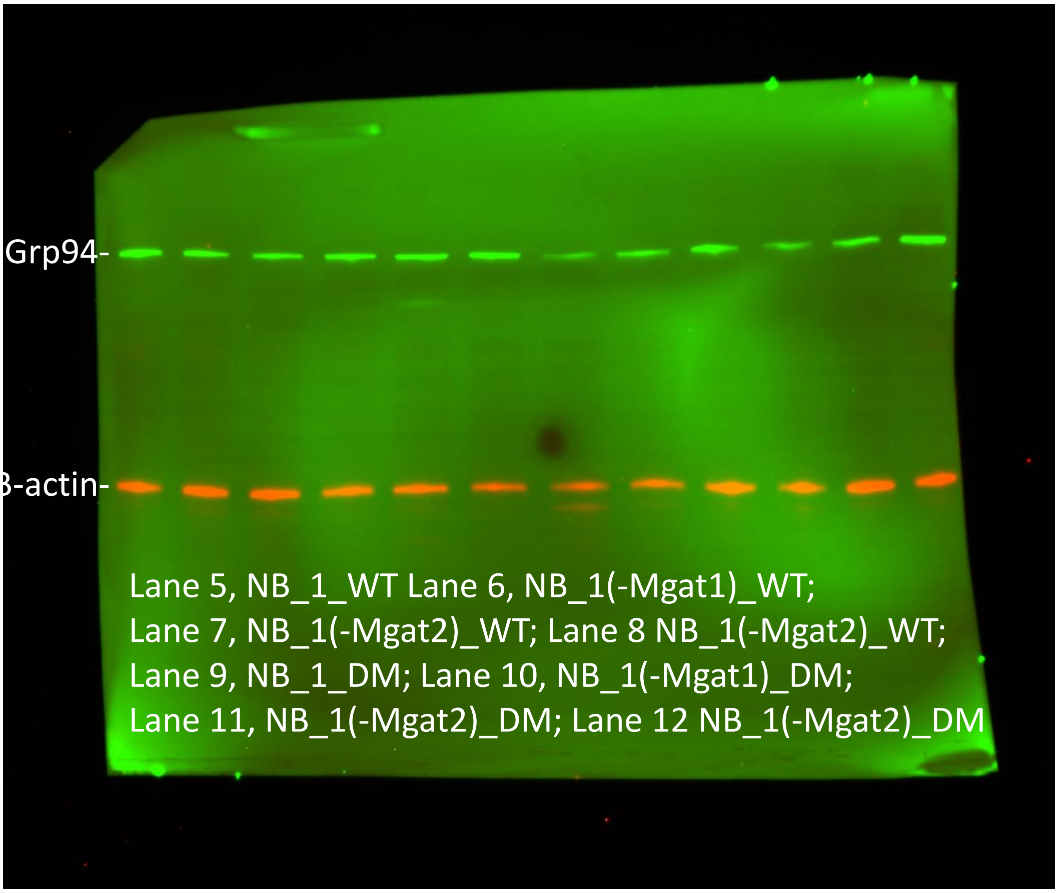

WT is WT Kv3.1b and DM is N220/229Q Kv3.1b

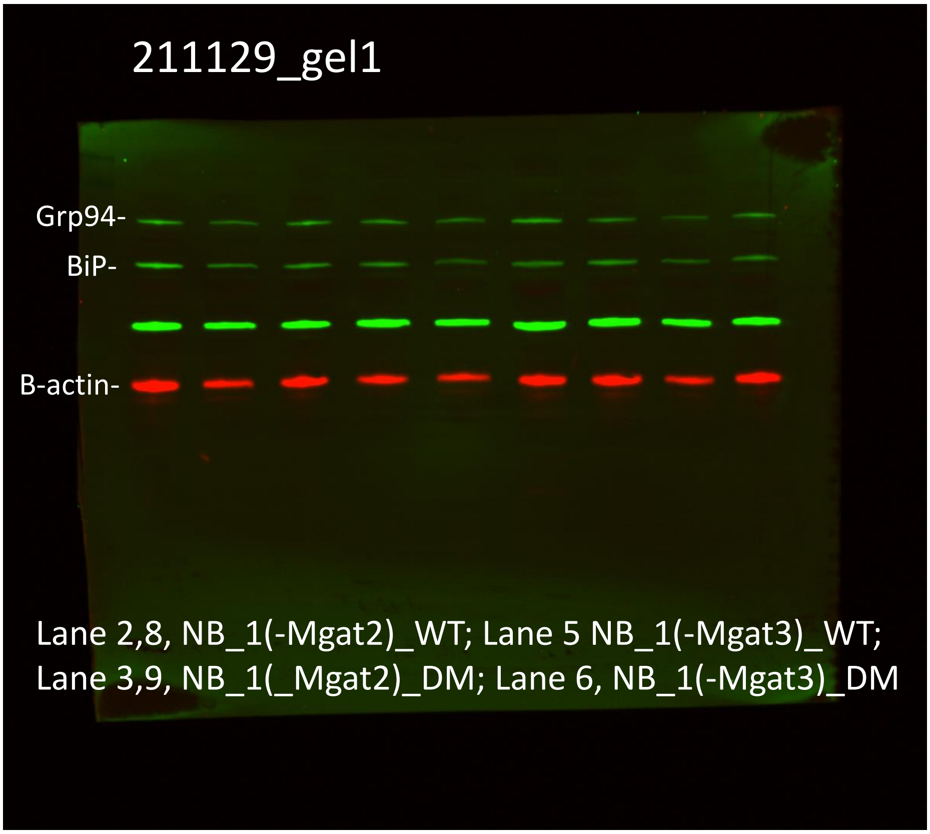
211129_gel1
Grp94-
BiP-
B-actin-
Lane 2,8, NB_1(-Mgat2)_WT; Lane 5 NB_1(-Mgat3)_WT;
Lane 3,9, NB_1(_Mgat2)_DM; Lane 6, NB_1(-Mgat3)_DM

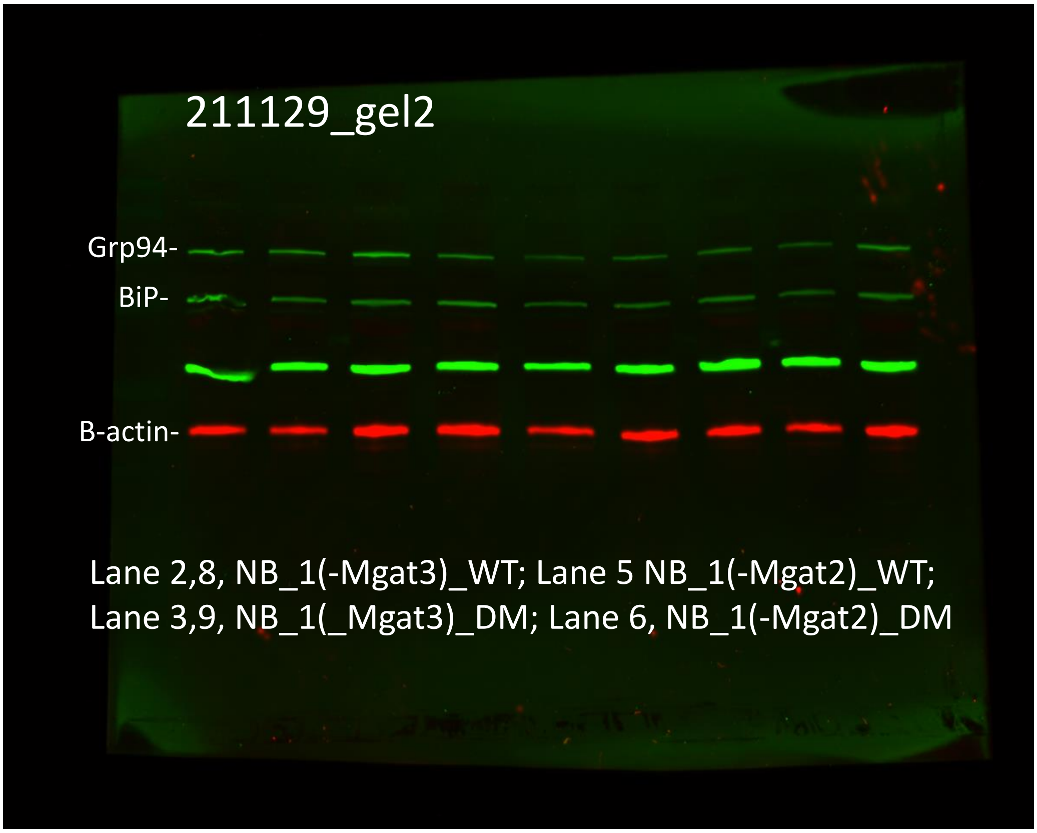
211129_gel2
Grp94-
BiP-
B-actin-
Lane 2,8, NB_1(-Mgat3)_WT; Lane 5 NB_1(-Mgat2)_WT;
Lane 3,9, NB_1(_Mgat3)_DM; Lane 6, NB_1(-Mgat2)_DM

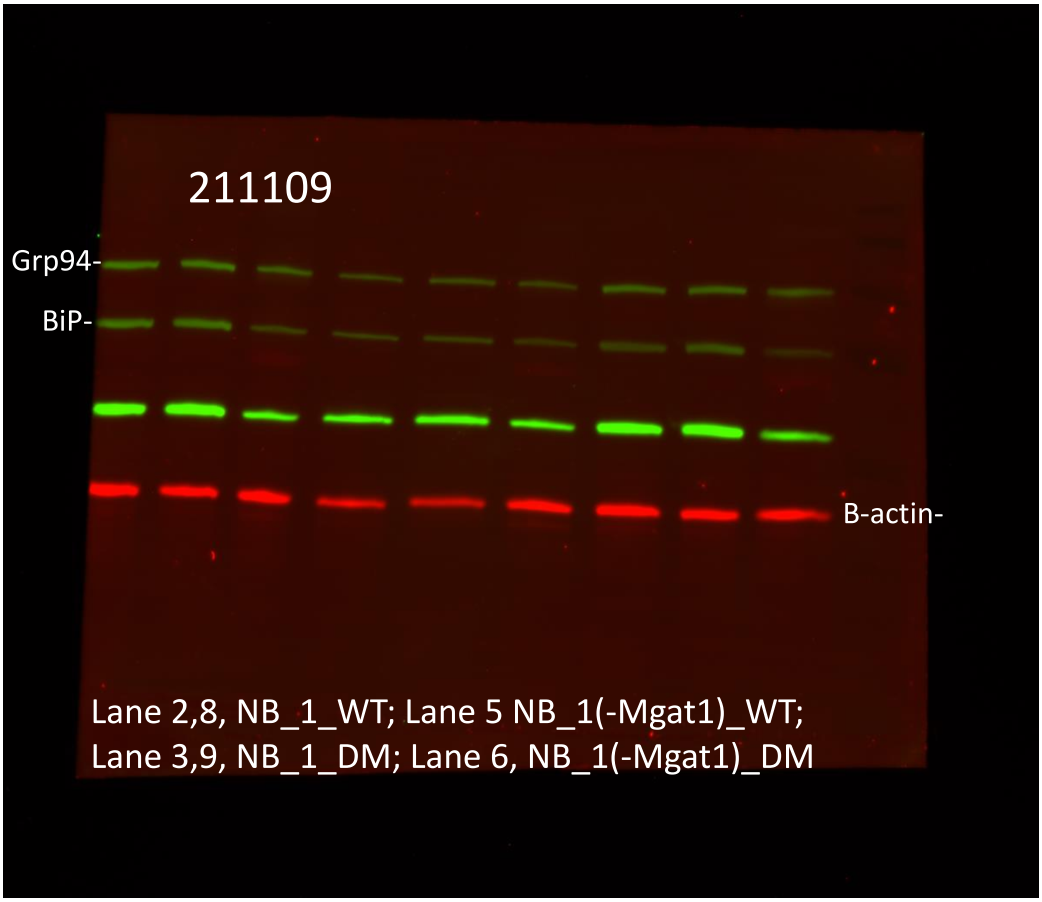
211109
Grp94-
BiP-
B-actin-
Lane 2,8, NB_1_WT; Lane 5 NB_1(-Mgat1)_WT;
Lane 3,9, NB_1_DM; Lane 6, NB_1(-Mgat1)_DM

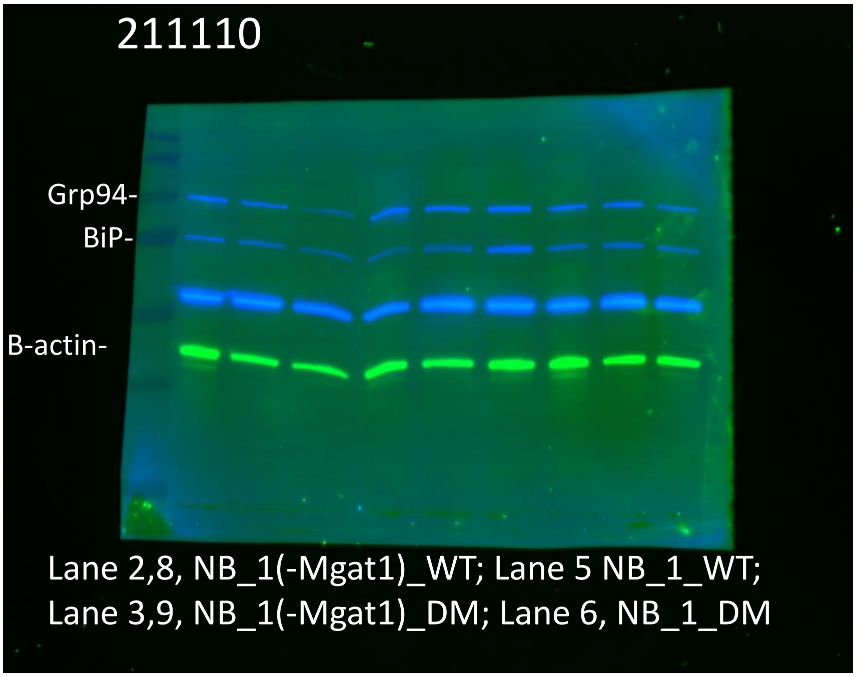
211110
Grp94-
BiP-
B-actin-
Lane 2,8, NB_1(-Mgat1)_WT; Lane 5 NB_1_WT;
Lane 3,9, NB_1(-Mgat1)_DM; Lane 6, NB_1_DM

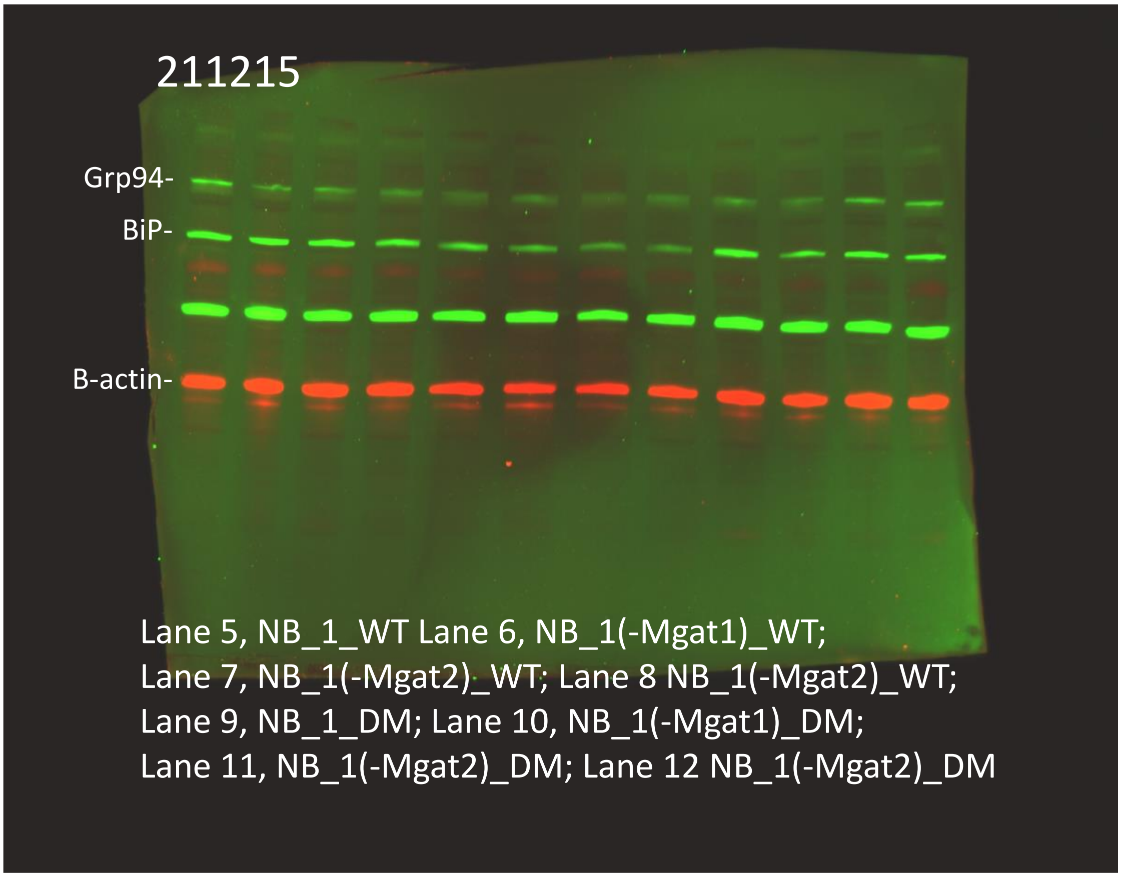
211215
Grp94-
BiP-
B-actin-
Lane 5, NB_1_WT Lane 6, NB_1(-Mgat1)_WT;
Lane 7, NB_1(-Mgat2)_WT; Lane 8 NB_1(-Mgat2)_WT;
Lane 9, NB_1_DM; Lane 10, NB_1(-Mgat1)_DM;
Lane 11, NB_1(-Mgat2)_DM; Lane 12 NB_1(-Mgat2)_DM

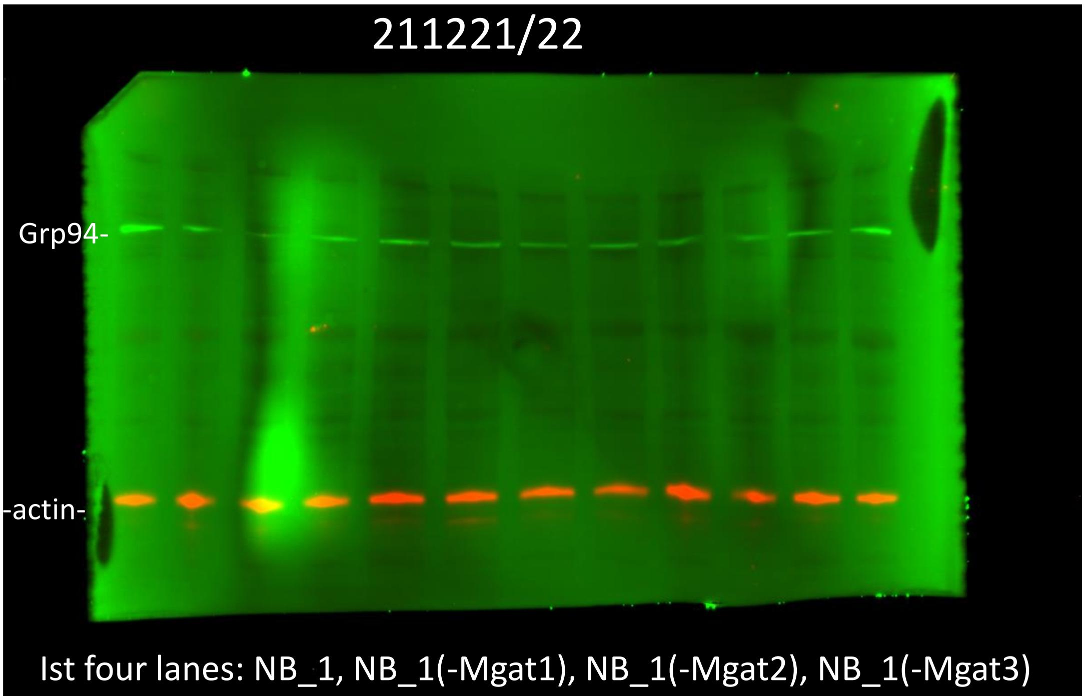
211221/22
Grp94-
-actin-
Ist four lanes: NB_1, NB_1(-Mgat1), NB_1(-Mgat2), NB_1(-Mgat3)

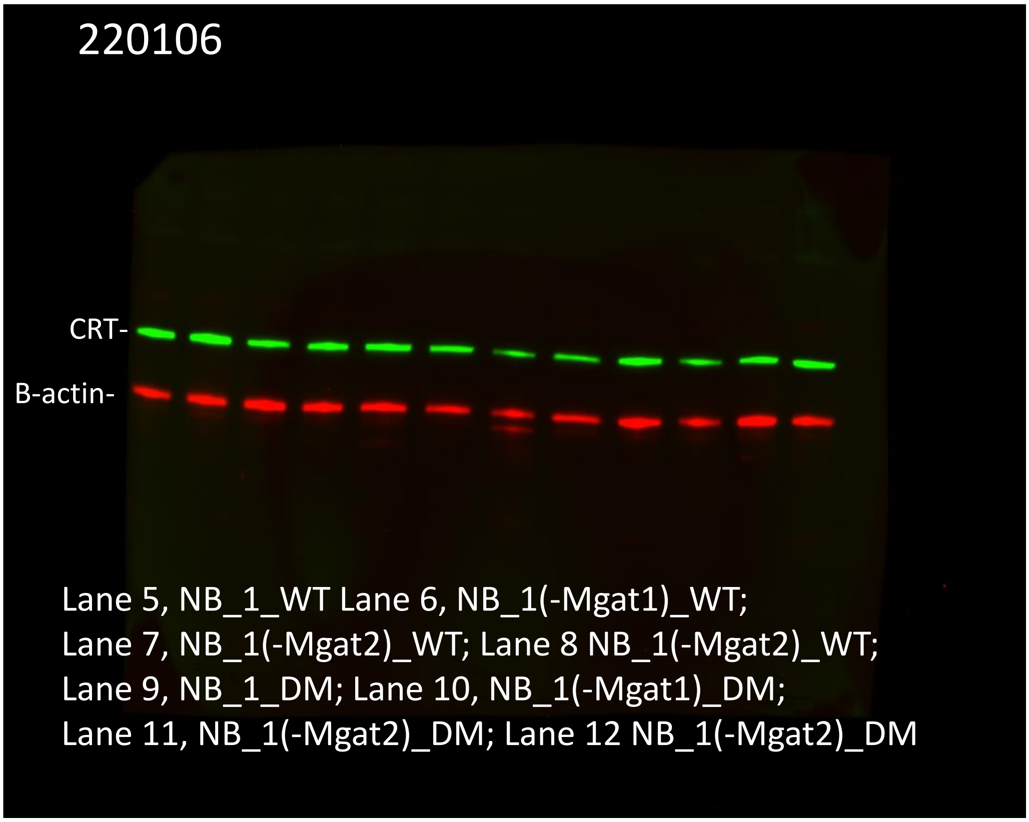
220106
CRT-
B-actin-
Lane 5, NB_1_WT Lane 6, NB_1(-Mgat1)_WT;
Lane 7, NB_1(-Mgat2)_WT; Lane 8 NB_1(-Mgat2)_WT;
Lane 9, NB_1_DM; Lane 10, NB_1(-Mgat1)_DM;
Lane 11, NB_1(-Mgat2)_DM; Lane 12 NB_1(-Mgat2)_DM

211215

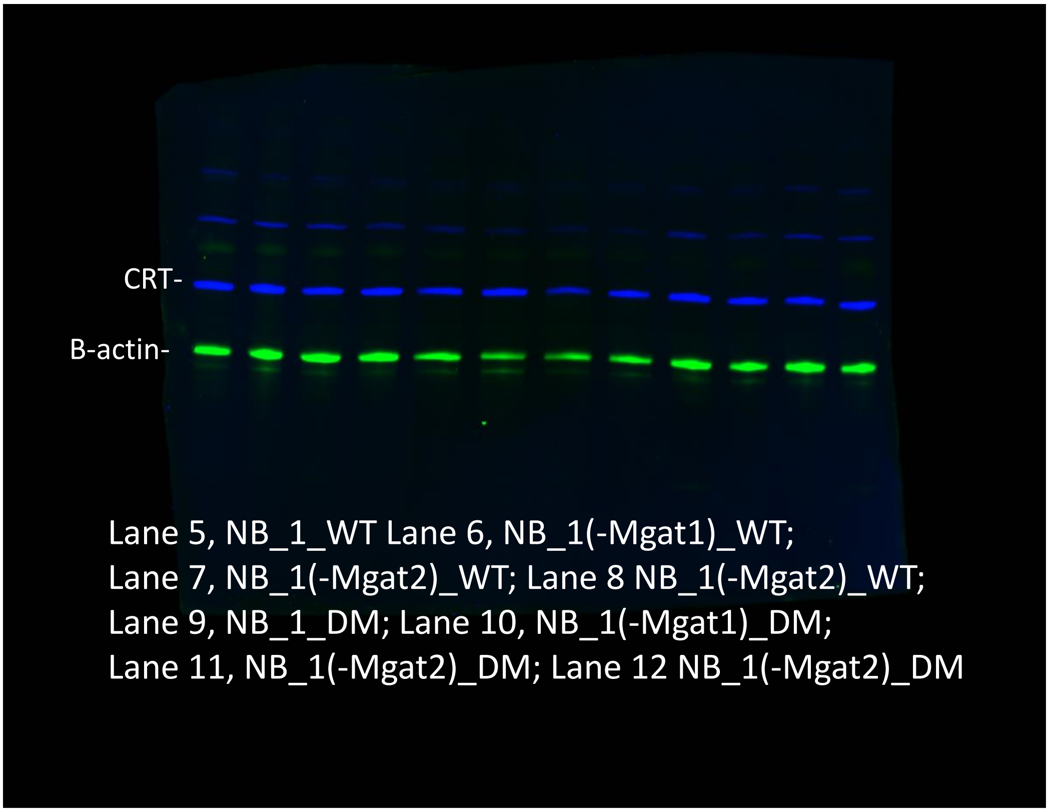
CRT-
B-actin-
Lane 5, NB_1_WT Lane 6, NB_1(-Mgat1)_WT;
Lane 7, NB_1(-Mgat2)_WT; Lane 8 NB_1(-Mgat2)_WT;
Lane 9, NB_1_DM; Lane 10, NB_1(-Mgat1)_DM;
Lane 11, NB_1(-Mgat2)_DM; Lane 12 NB_1(-Mgat2)_DM

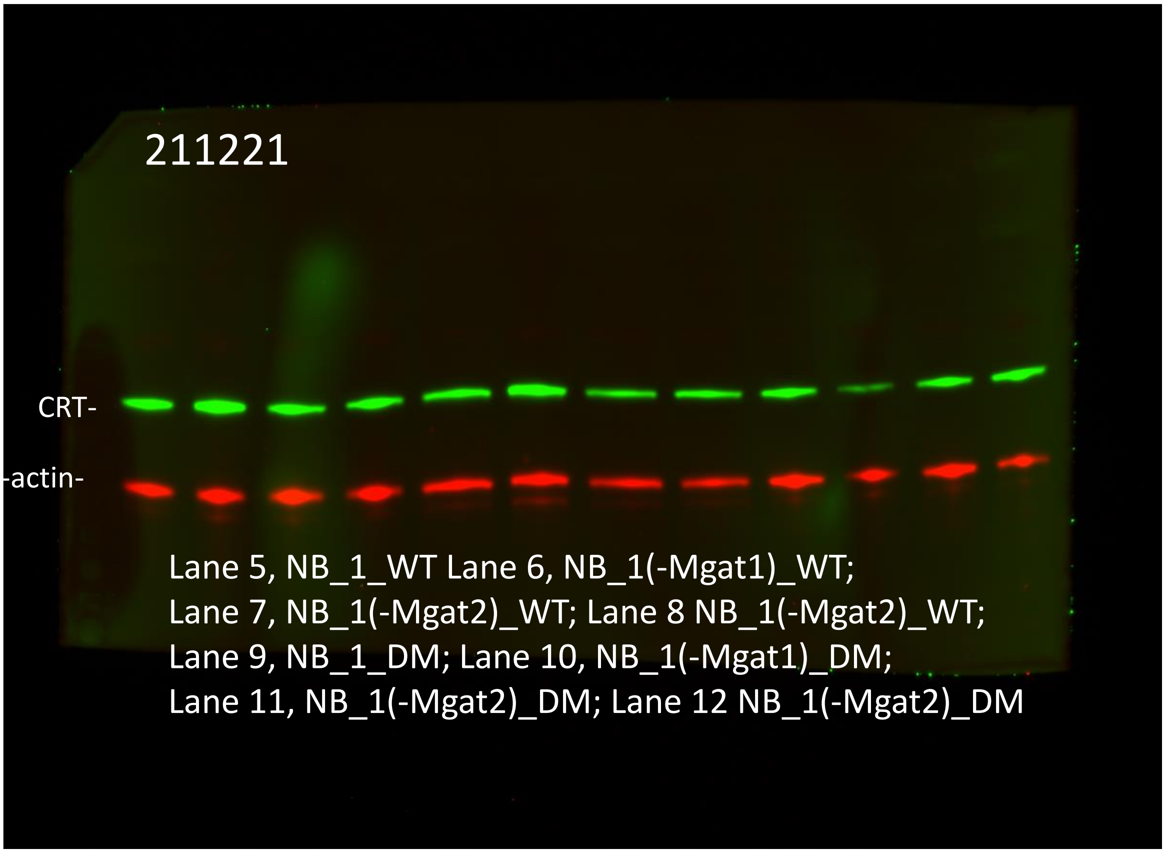
211221
CRT-
-actin-
Lane 5, NB_1_WT Lane 6, NB_1(-Mgat1)_WT;
Lane 7, NB_1(-Mgat2)_WT; Lane 8 NB_1(-Mgat2)_WT;
Lane 9, NB_1_DM; Lane 10, NB_1(-Mgat1)_DM;
Lane 11, NB_1(-Mgat2)_DM; Lane 12 NB_1(-Mgat2)_DM

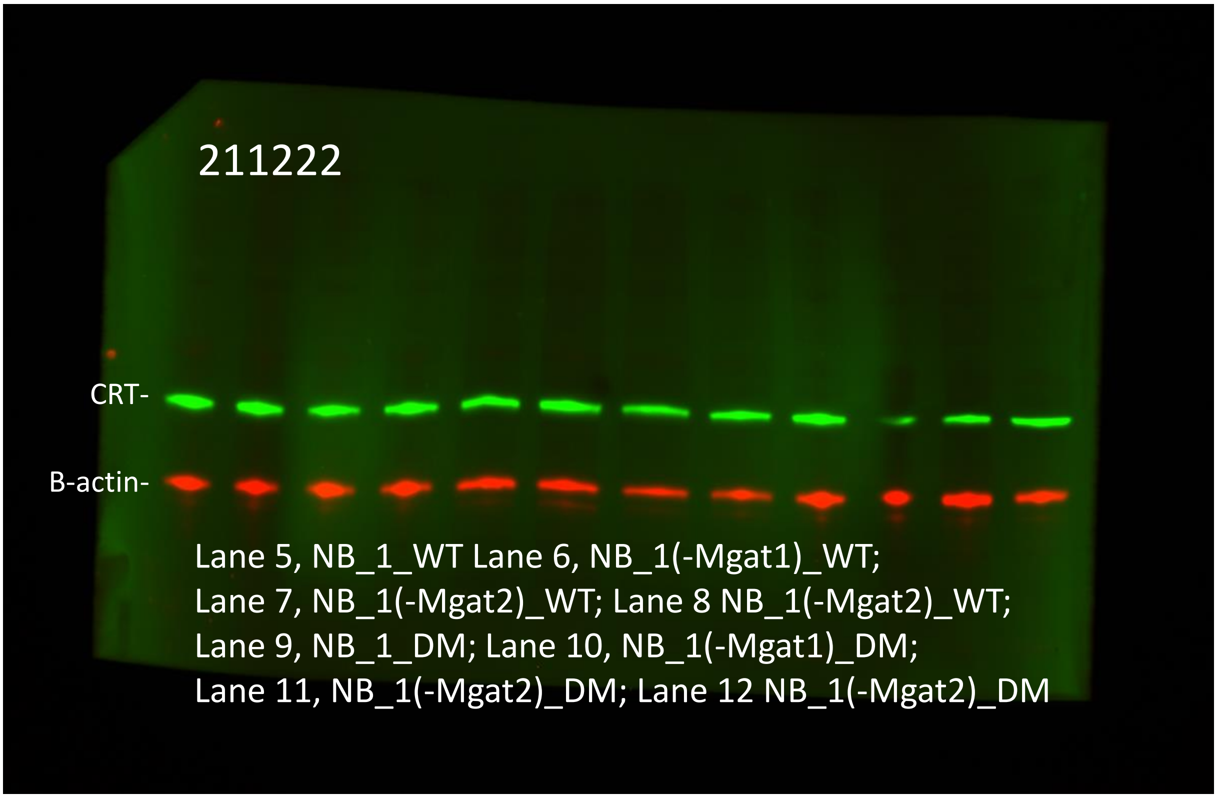
211222
CRT-
B-actin-
Lane 5, NB_1_WT Lane 6, NB_1(-Mgat1)_WT;
Lane 7, NB_1(-Mgat2)_WT; Lane 8 NB_1(-Mgat2)_WT;
Lane 9, NB_1_DM; Lane 10, NB_1(-Mgat1)_DM;
Lane 11, NB_1(-Mgat2)_DM; Lane 12 NB_1(-Mgat2)_DM

Western Blot analysis via Image Lab (Bio-Rad)	Neurofilament (NF-L)	NF-L Intensity
	220216	8533780
		882198
		4078329
		12111297
		1354352
		4870774
		2170774
		11349180
	220315	7571042
		192136
		3044261
		15612048
		2038605
		7748136
		289341
		19633189
	220316	7747198
		94640
		2975965
		15565812
		1998648
		7202384
		282374
		19634606
	220321	10304460
		543144
		3396536
		13354536
		553761
		5409392
		1746682
		13634880

β-actin Intensity	NF-L/β-actin	Samples	Vimentin (vim)	Vim Intensity
18598010	0.458854469	NB_1	220127	17935430
18944630	0.04656718	NB_1(-Mgat1)		22543880
24232265	0.168301601	NB_1(-Mgat2)		6818435
20994336	0.576884022	NB_1(-Mgat3)		32242412
24658992	0.054923251	NB_1_wt Kv3.1b		1712445
17671995	0.27562106	NB_1(-Mgat1)_wt Kv3.1b		19212693
24212732	0.089654236	NB_1(-Mgat2)_wt Kv3.1b		9060774
15248980	0.744258304	NB_1(-Mgat3)_wt Kv3.1b		8859730
35117554	0.215591382	NB_1	220201_1	25825646
37387278	0.005139074	NB_1(-Mgat1)		34502685
51311874	0.059328587	NB_1(-Mgat2)		8617238
44278512	0.352587458	NB_1(-Mgat3)		54083007
40214120	0.050693761	NB_1_wt Kv3.1b		889440
39785850	0.194746022	NB_1(-Mgat1)_wt Kv3.1b		22922615
36835500	0.00785495	NB_1(-Mgat2)_wt Kv3.1b		6976908
26973140	0.727879253	NB_1(-Mgat3)_wt Kv3.1b		9158930
35334694	0.219251877	NB_1	220201_2	45596390
37400908	0.00253042	NB_1(-Mgat1)		32034095
51316010	0.057992915	NB_1(-Mgat2)		7862485
43774296	0.355592515	NB_1(-Mgat3)		27240300
40194120	0.049724885	NB_1_wt Kv3.1b		1464900
39959696	0.180241211	NB_1(-Mgat1)_wt Kv3.1b		25464180
36695168	0.007695128	NB_1(-Mgat2)_wt Kv3.1b		9924935
26981969	0.727693594	NB_1(-Mgat3)_wt Kv3.1b		11694500
27785505	0.370857395	NB_1		
30369885	0.017884296	NB_1(-Mgat1)		
36418345	0.093264425	NB_1(-Mgat2)		
30169854	0.442645032	NB_1(-Mgat3)		
31928832	0.017343603	NB_1_wt Kv3.1b		
30260986	0.178757956	NB_1(-Mgat1)_wt Kv3.1b		
33690804	0.051844474	NB_1(-Mgat2)_wt Kv3.1b		
24956400	0.546348031	NB_1(-Mgat3)_wt Kv3.1b		

β-actin Intensity	Vim/β-actin	Sample	BiP		BiP Intensity
25405375	0.7059699	NB_1		211109	21189408
28769040	0.783616	NB_1(-Mgat1)			21498070
33538040	0.20330452	NB_1(-Mgat2)			8385260
33728832	0.95593029	NB_1(-Mgat3)			9202340
29883767	0.05730352	NB_1_wt Kv3.1b			6140958
26761769	0.71791566	NB_1(-Mgat1)_wt Kv3.1b			15191070
34475056	0.26282115	NB_1(-Mgat2)_wt Kv3.1b		211110	23151570
21624706	0.40970407	NB_1(-Mgat3)_wt Kv3.1b			19901310
32703356	0.78969406	NB_1			16998955
31631215	1.09077963	NB_1(-Mgat1)			19799716
36429514	0.23654551	NB_1(-Mgat2)			43930821
33299936	1.62411745	NB_1(-Mgat3)			24484420
37459392	0.02374411	NB_1_wt Kv3.1b		211215	25118950
34598145	0.66253884	NB_1(-Mgat1)_wt Kv3.1b			22330792
39273808	0.17764786	NB_1(-Mgat2)_wt Kv3.1b			23206600
22840168	0.40100099	NB_1(-Mgat3)_wt Kv3.1b			16520200
32750395	1.39223939	NB_1(-Mgat3)			16052568
37980210	0.84344176	NB_1(-Mgat1)			11535614
37255581	0.2110418	NB_1(-Mgat2)			11316348
31176834	0.87373529	NB_1			8350800
37013174	0.0395778	NB_1_wt Kv3.1b		220110	24627456
33181056	0.76743127	NB_1(-Mgat1)_wt Kv3.1b			24713100
37718383	0.26313257	NB_1(-Mgat2)_wt Kv3.1b			25035936
27773366	0.42106888	NB_1(-Mgat3)_wt Kv3.1b			18529359
					31283545
					14498440
					9894780
					9950679
			211129_gel1		5496288
					2501632
					2883104
					1897740
					3809568
					1742832
			211129_gel2		23015275
					14651015
					22411403
					11841039
					17826089
					12391128

β-actin Intensity	BiP/β-actin	Sample	CRT	CRT Intensity
39756934	0.532973896	NB_1	211215	31326440
33586670	0.640077447	NB_1_wt Kv3.1b		33087436
25237170	0.332258332	NB_1(-Mgat1)		21606934
24542386	0.374957023	NB_1(-Mgat1)_wt Kv3.1b		25163612
44256254	0.138759101	NB_1		15346998
34228255	0.443816666	NB_1_wt Kv3.1b		22203330
45543069	0.50834453	NB_1(-Mgat1)		9098300
25009448	0.79575167	NB_1(-Mgat1)_wt Kv3.1b		14707804
37494054	0.453377354	NB_1	211221	25837014
27903084	0.709588804	NB_1_wt Kv3.1b		40323570
49752144	0.882993525	NB_1(-Mgat1)		24378068
28189629	0.868561271	NB_1(-Mgat1)_wt Kv3.1b		23619822
27490815	0.913721547	NB_1		23066603
30352432	0.735716729	NB_1(-Mgat1)		33519024
33374165	0.695346236	NB_1(-Mgat2)		16522965
31408600	0.525976962	NB_1(-Mgat3)		18408708
23960640	0.669955727	NB_1_wt Kv3.1b	211222	31896510
15511328	0.743689644	NB_1(-Mgat1)_wt Kv3.1b		32066648
16155271	0.700474043	NB_1(-Mgat2)_wt Kv3.1b		28621596
19537100	0.427432935	NB_1(-Mgat3)_wt Kv3.1b		31002696
19085568	1.290370609	NB_1		27083056
26637024	0.927772562	NB_1(-Mgat1)		34243944
33966958	0.737067358	NB_1(-Mgat2)		23751792
25894330	0.71557592	NB_1(-Mgat3)		26189460
25873497	1.209096126	NB_1_wt Kv3.1b	220106	31032974
22362494	0.648337346	NB_1(-Mgat1)_wt Kv3.1b		44441210
19019160	0.52025326	NB_1(-Mgat2)_wt Kv3.1b		24642999
20628174	0.482382929	NB_1(-Mgat3)_wt Kv3.1b		26857600
12357115	0.444787315	NB_1(-Mgat2)		29058003
5866244.333	0.426445245	NB_1(-Mgat2)_wt Kv3.1b		21327412
6924489.333	0.416363411	NB_1(-Mgat3)		12663750
4341375	0.43712879	NB_1(-Mgat3)_wt Kv3.1b		16238502
10026490	0.379950312	NB_1(-Mgat2)		
4799788	0.363106037	NB_1(-Mgat2)_wt Kv3.1b		
24739994	0.9302862	NB_1(-Mgat3)		
18156388	0.806934452	NB_1(-Mgat3)_wt Kv3.1b		
39923856	0.561353668	NB_1(-Mgat2)		
21207912	0.558331202	NB_1(-Mgat2)_wt Kv3.1b		
29325534	0.607869204	NB_1(-Mgat3)		
20812566	0.595367626	NB_1(-Mgat3)_wt Kv3.1b		

β-actin Intensity	CRT/β-actin	Sample	Grp94	Grp94 Intensity
27651510	1.132901603	NB_1	211215	10989631
30095510	1.099414364	NB_1(-Mgat1)		8019060
33532808	0.644352063	NB_1(-Mgat2)		7030609
31393180	0.801563015	NB_1(-Mgat3)		9357922
23972144	0.64020131	NB_1_wt Kv3.1b		6845668
15593788	1.423857372	NB_1(-Mgat1)_wt Kv3.1b		7446625
16143881	0.56357576	NB_1(-Mgat2)_wt Kv3.1b	211110	
19532799	0.752979847	NB_1(-Mgat3)_wt Kv3.1b		36210050
23655338	1.09222764	NB_1		22723968
27611402	1.4603956	NB_1(-Mgat1)		47836846
29639708	0.822480033	NB_1(-Mgat2)		37217250
28434700	0.830668936	NB_1(-Mgat3)		30995030
29288031	0.787577799	NB_1_wt Kv3.1b		35455305
28449680	1.178186328	NB_1(-Mgat1)_wt Kv3.1b	211109	20425836
22620408	0.730445048	NB_1(-Mgat2)_wt Kv3.1b		19615422
18929206	0.972502914	NB_1(-Mgat3)_wt Kv3.1b		9002370
25192904	1.266091039	NB_1		10682000
24858412	1.289971701	NB_1(-Mgat1)		15862530
29373312	0.974408197	NB_1(-Mgat2)	211221	18116493
30272640	1.024116033	NB_1(-Mgat3)		7211428
27994896	0.967428348	NB_1_wt Kv3.1b		9426015
28248000	1.212260833	NB_1(-Mgat1)_wt Kv3.1b		14183171
21605280	1.09935127	NB_1(-Mgat2)_wt Kv3.1b		7266270
21362232	1.225970208	NB_1(-Mgat3)_wt Kv3.1b	211119 gel1	3424350
28188672	1.100902306	NB_1		2289456
35430912	1.254306127	NB_1(-Mgat1)		2611719
45856368	0.537395352	NB_1(-Mgat2)		1547640
35523773	0.756045818	NB_1(-Mgat3)	211119 gel2	20700690
37341444	0.778170309	NB_1_wt Kv3.1b		17175710
26831876	0.794853554	NB_1(-Mgat1)_wt Kv3.1b		12462496
22898393	0.553040993	NB_1(-Mgat2)_wt Kv3.1b		14842820
28615254	0.567477123	NB_1(-Mgat3)_wt Kv3.1b		10472015

β-actin Intensity	Grp94/β-actin	Sample	KIF5B	KIF Intensity
27490815	0.399756464	NB_1	211116	28710000
33374165	0.240277472	NB_1(-Mgat2)		15688420
23960640	0.293423256	NB_1_wt Kv3.1b		22106994
15511328	0.603295991	NB_1(-Mgat1)_wt Kv3.1b		35561878
16155271	0.423742072	NB_1(-Mgat2)_wt Kv3.1b		27964108
19537100	0.381153037	NB_1(-Mgat3)_wt Kv3.1b		17153400
			211108	5632608
90926820	0.398232887	NB_1(-Mgat1)		6636320
50028748	0.454218203	NB_1(-Mgat1)_wt Kv3.1b		11103576
75575470	0.632967893	NB_1		18160920
55391644	0.671892858	NB_1_wt Kv3.1b		9124060
99214634	0.312403813	NB_1(-Mgat1)		14410640
56100680	0.631994211	NB_1(-Mgat1)_wt Kv3.1b	211130_gel 1	31769046
39756934	0.513767888	NB_1		18253003
33586670	0.584024019	NB_1_wt Kv3.1b		17290428
25237170	0.356710756	NB_1(-Mgat1)		11633066
24542386	0.435247005	NB_1(-Mgat1)_wt Kv3.1b		26526298
34228255	0.463433792	NB_1_wt Kv3.1b		15826441
25433952	0.712295635	NB_1	211130_gel 2	21146255
28143744	0.25623556	NB_1(-Mgat1)		10420634
33595008	0.280577847	NB_1(-Mgat2)		28612260
32578925	0.435348036	NB_1_wt Kv3.1b		16353699
20951259	0.346817821	NB_1(-Mgat3)_wt Kv3.1b		15430941
12376597.33	0.276679438	NB_1(-Mgat2)		9401826
5876192	0.389615588	NB_1(-Mgat2)_wt Kv3.1b	220106	8456235
6921002.667	0.377361363	NB_1(-Mgat3)		10489900
4934869.333	0.313613167	NB_1(-Mgat2)_wt Kv3.1b		25233852
24735720	0.836874366	NB_1(-Mgat3)		9358260
39933909	0.430103399	NB_1(-Mgat2)		19284474
21218176	0.587350015	NB_1(-Mgat2)_wt Kv3.1b		17302221
29326614	0.506121164	NB_1(-Mgat3)		16694544
20818908	0.503005009	NB_1(-Mgat3)_wt Kv3.1b		12489155

β-actin Intensity	Kif/β-actin	Sample
70768096	0.40569129	NB_1(-Mgat1)
29571808	0.53051947	NB_1(-Mgat1)_wt Kv3.1b
56903040	0.38850286	NB_1
50951628	0.69795371	NB_1_wt Kv3.1b
66582656	0.41999088	NB_1(-Mgat1)
34308288	0.49997831	NB_1(-Mgat1)_wt Kv3.1b
15198608	0.37060025	NB_1(-Mgat1)
16254189	0.40828367	NB_1(-Mgat1)_wt Kv3.1b
22275652	0.49846245	NB_1
21579880	0.84156724	NB_1_wt Kv3.1b
22375360	0.40777266	NB_1
21569234	0.66811088	NB_1_wt Kv3.1b
39929526	0.79562793	NB_1(-Mgat2)
20084862	0.90879405	NB_1(-Mgat2)_wt Kv3.1b
31794355	0.54382069	NB_1(-Mgat3)
20701980	0.56193011	NB_1(-Mgat3)_wt Kv3.1b
34469952	0.76954845	NB_1(-Mgat2)
17418612	0.90859369	NB_1(-Mgat2)_wt Kv3.1b
35443170	0.59662426	NB_1(-Mgat3)
21416496	0.48657045	NB_1(-Mgat3)_wt Kv3.1b
27813890	1.028704	NB_1(-Mgat2)
20301375	0.80554637	NB_1(-Mgat2)_wt Kv3.1b
32842374	0.46984853	NB_1(-Mgat3)
22218600	0.42315114	NB_1(-Mgat3)_wt Kv3.1b
22899560	0.369275	NB_1
32322515	0.32453848	NB_1(-Mgat1)
35910950	0.70267849	NB_1(-Mgat2)
23099630	0.40512597	NB_1(-Mgat3)
35474450	0.54361587	NB_1_wt Kv3.1b
35155232	0.49216632	NB_1(-Mgat1)_wt Kv3.1b
23705360	0.70425187	NB_1(-Mgat2)_wt Kv3.1b
30091323	0.41504174	NB_1(-Mgat3)_wt Kv3.1b
