# Supplementary material for: Kv3-Expressing Cells Present More Elaborate N-Glycans with Changes in Cytoskeletal Proteins, Neurite Structure and Cell Migration
Source: COJ Biomed Sci Res. Author manuscript; Available in PMC 2024 Dec 30. (PMC11684427)
Supplement: supp figures/tables [file NIHMS2040166-supplement-supp_figures_tables.pdf]

A.

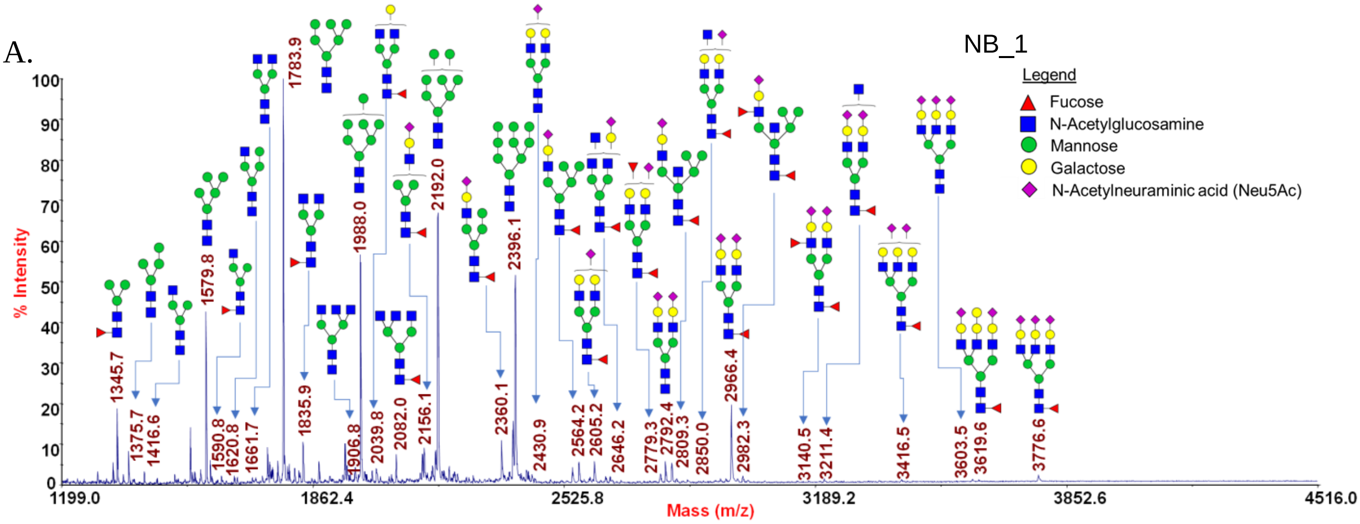

B.

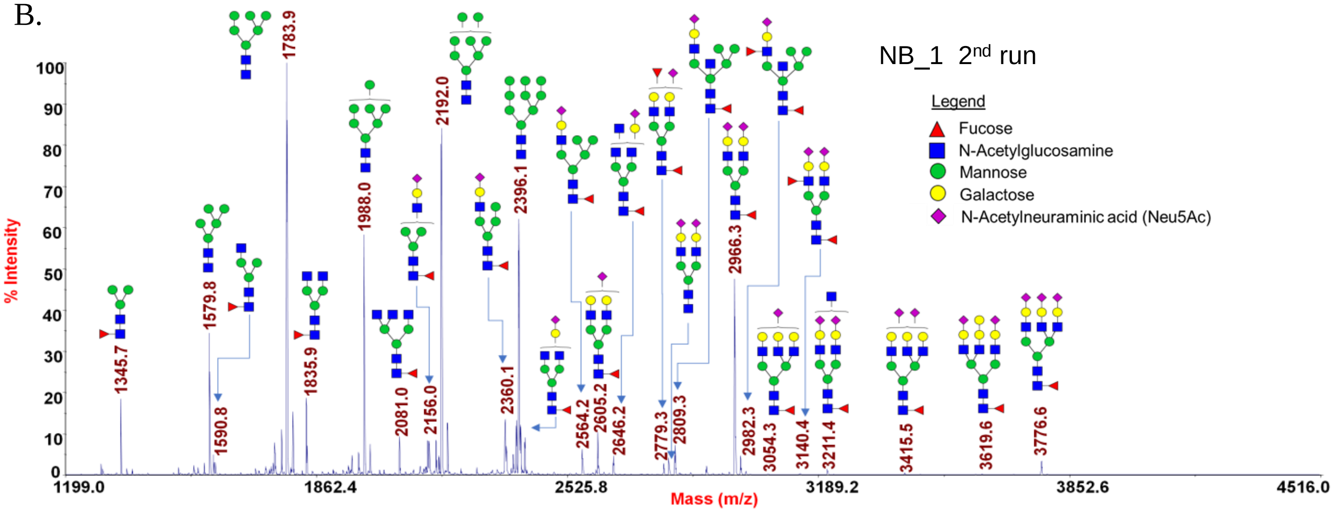

Fig S1. MALDI-TOF MS spectrum of the permethylated N-glycans derived from the NB_1 cell line which was obtained in parallel to the spectra acquired for NB cell lines expressing Wt Kv3.1b, see Fig 3, (A) and an additional spectrum of the glycans from NB_1 cell line (B).

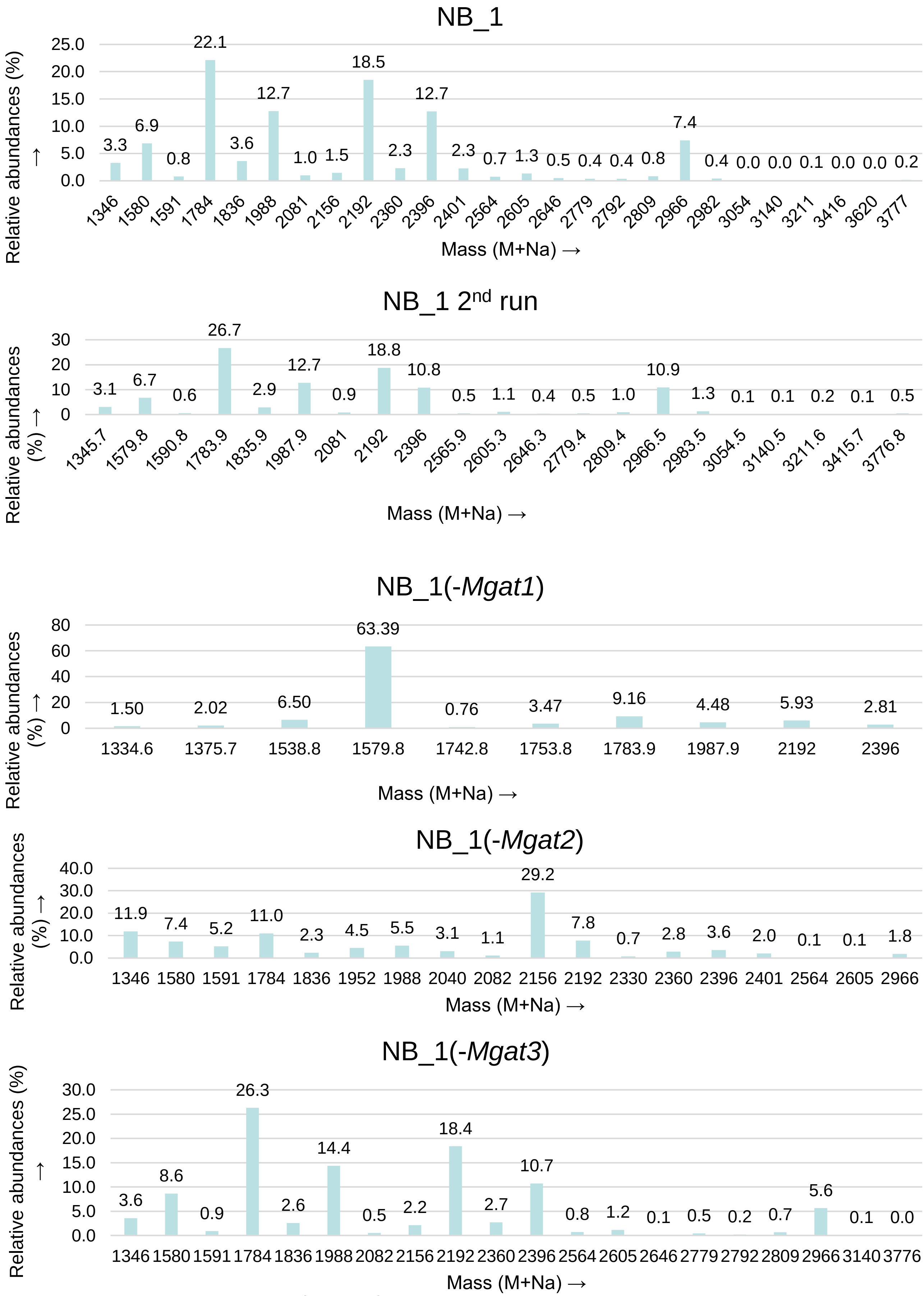

Fig S2. Relative abundancies of each of the N-glycan structures shown on the MALDI-TOF MS profiles from NB_1, NB_1 2nd run, NB_1(-*Mgat1*), NB_1(-*Mgat2*), and NB_1(-*Mgat3*). The percent of N-glycans shown in Figure 3D reports the average abundancies from all three runs of NB_1.

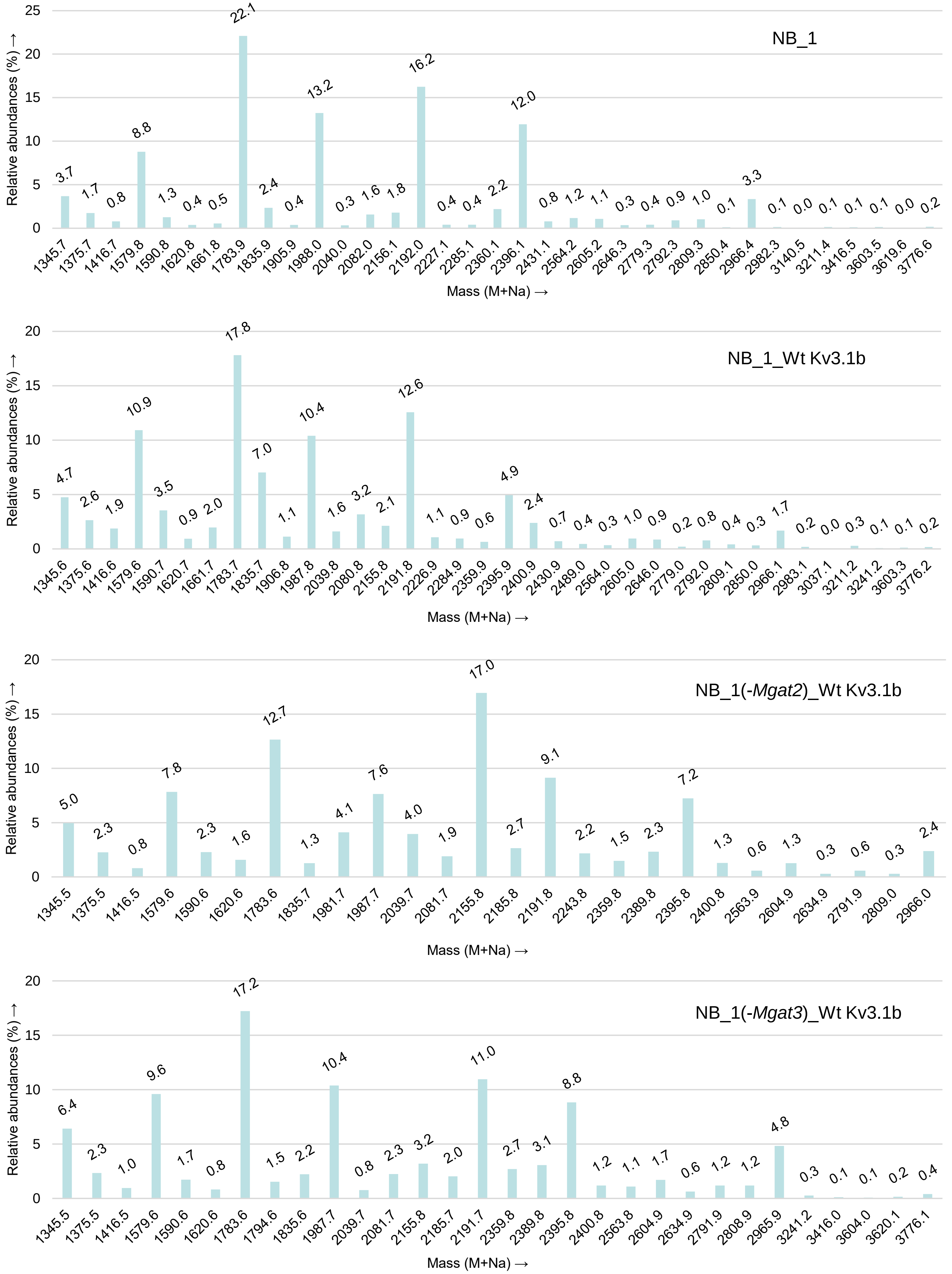

Fig S3. Relative levels of permethylated N-glycans derived from NB_1, NB_1(-*Mgat2*) and NB_1(-*Mgat3*) cell lines stably expressing Wt Kv3.1b. NB_1 cell line compared to NB_1 cells.

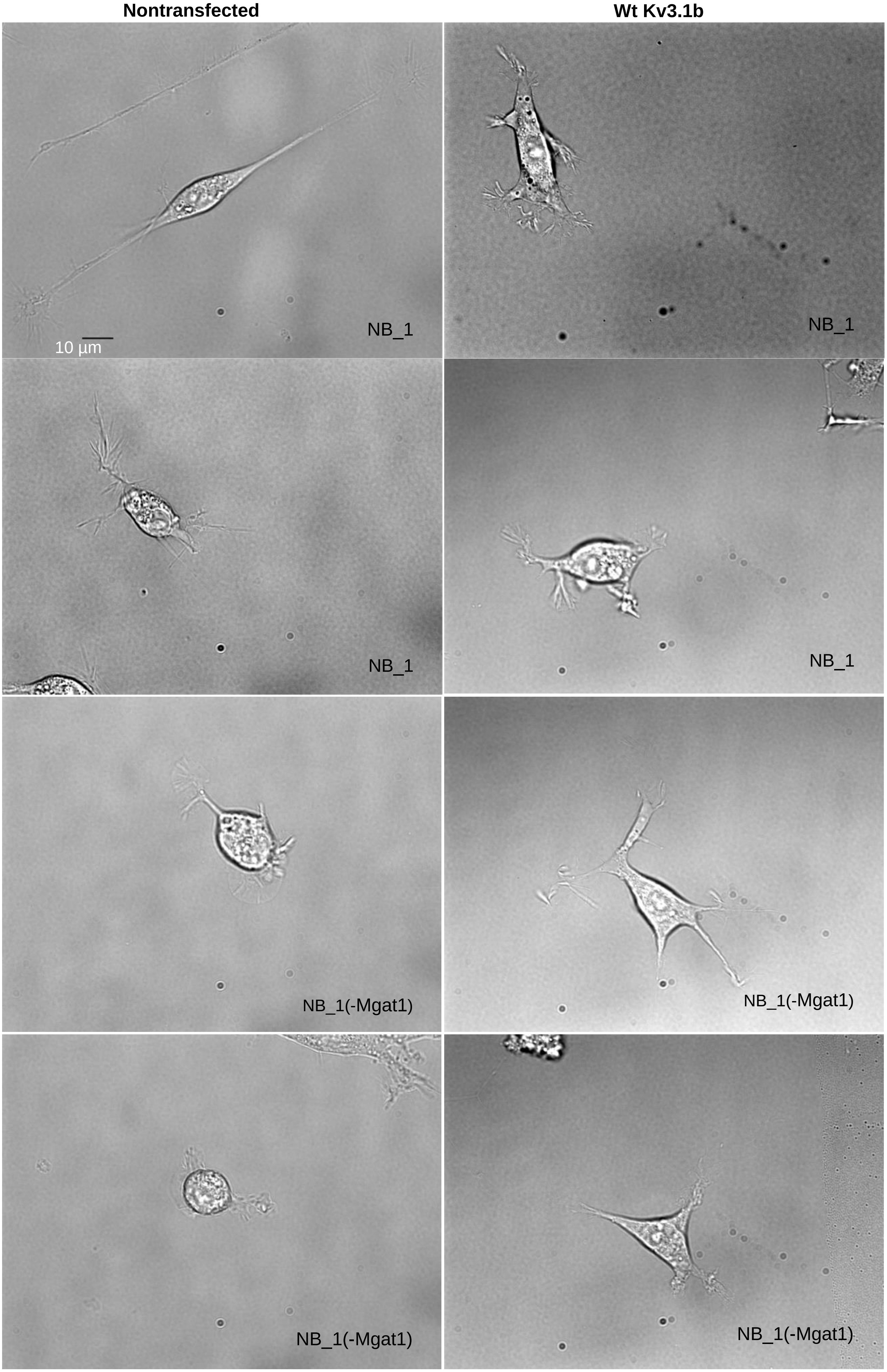

Fig S4. Cell morphology of NB_1 and NB_1(-*Mgat1*) cell lines and those stably expressing Wt Kv3.1b.

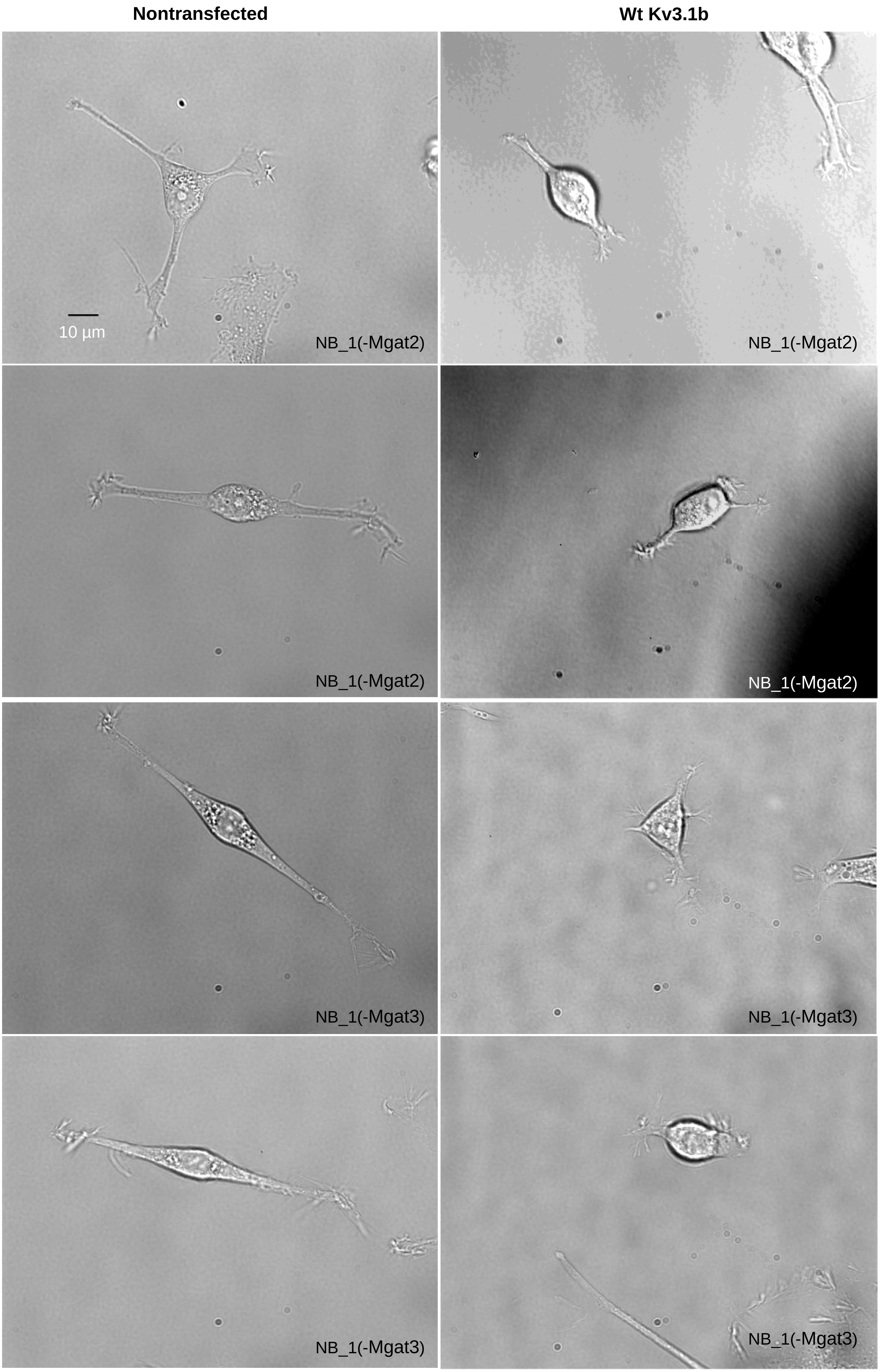

Fig S5. Cell morphology of NB_1(-*Mgat2*) and NB_1(-*Mgat3*) cell lines and those stably expressing Wt Kv3.1b.

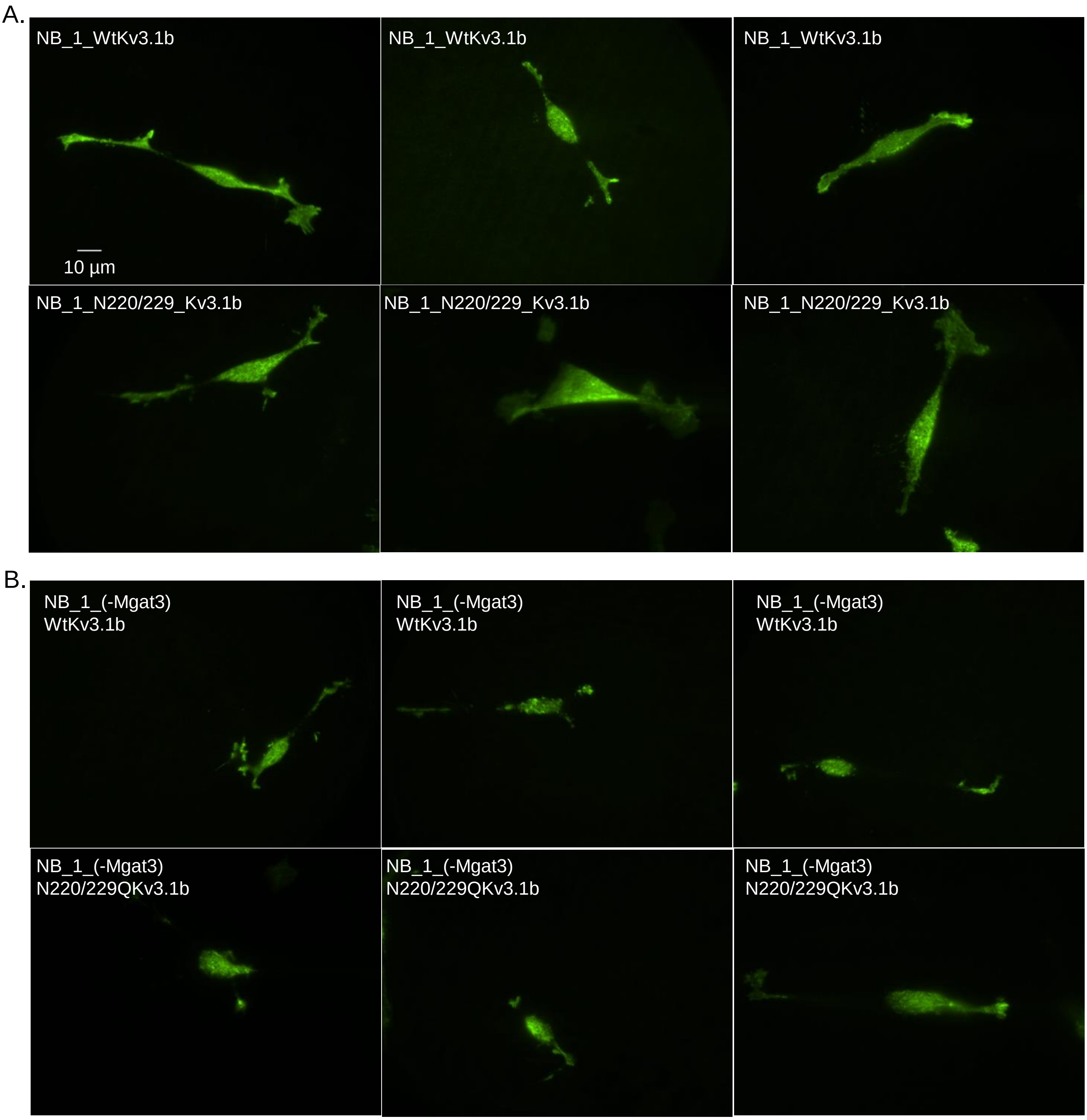

Fig S6. TIRF microscopy images of NB_1 (A) and NB_1(-*Mgat3*) (B) cell lines stably expressing Wt or N220/229Q Kv3.1b. All images are of similar size.

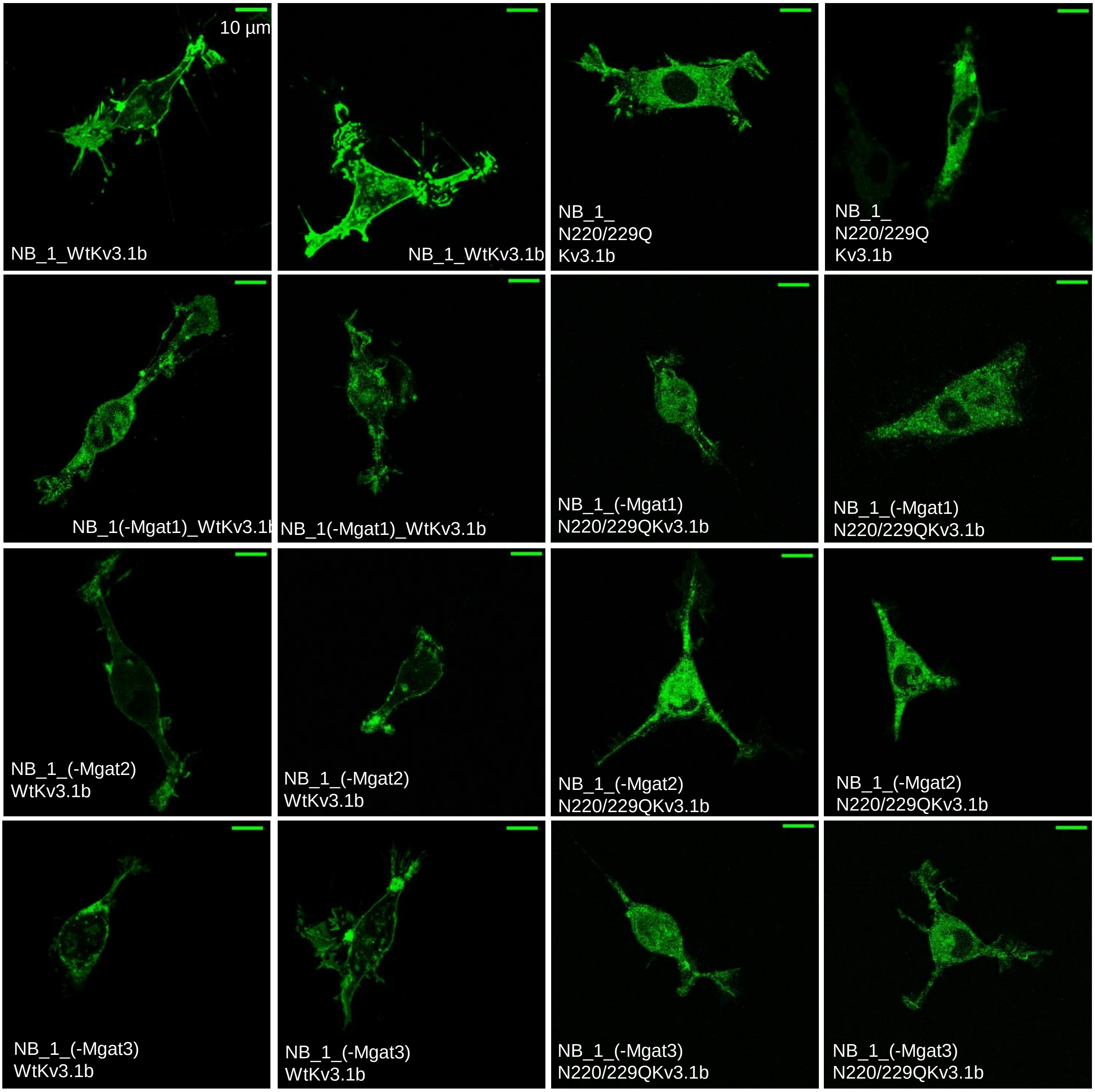

Fig S7. Confocal microscopy images of NB_1, NB_1(-*Mgat1*) , NB_1(-*Mgat2*), and NB_1(-*Mgat3*) cell lines stably expressing Wt or N220/229Q Kv3.1b. Scale bars, 10 µm.
